# From QTLs to Adaptation Landscapes: Using Genotype-To-Phenotype Models to Characterize G×E Over Time

**DOI:** 10.3389/fpls.2019.01540

**Published:** 2019-12-04

**Authors:** Daniela Bustos-Korts, Marcos Malosetti, Karine Chenu, Scott Chapman, Martin P. Boer, Bangyou Zheng, Fred A. van Eeuwijk

**Affiliations:** ^1^Biometris, Wageningen University and Research Centre, Wageningen, Netherlands; ^2^Queensland Alliance for Agriculture and Food Innovation, The University of Queensland, Toowoomba, QLD, Australia; ^3^Agriculture and Food, CSIRO, Queensland Bioscience Precinct, St Lucia, QLD, Australia; ^4^School of Agriculture and Food Sciences, The University of Queensland, Gatton, QLD, Australia

**Keywords:** QTL (quantitative trait loci), crop growth model, adaptation, G×E interaction, wheat, APSIM model, reaction norm

## Abstract

Genotype by environment interaction (G×E) for the target trait, e.g. yield, is an emerging property of agricultural systems and results from the interplay between a hierarchy of secondary traits involving the capture and allocation of environmental resources during the growing season. This hierarchy of secondary traits ranges from basic traits that correspond to response mechanisms/sensitivities, to intermediate traits that integrate a larger number of processes over time and therefore show a larger amount of G×E. Traits underlying yield differ in their contribution to adaptation across environmental conditions and have different levels of G×E. Here, we provide a framework to study the performance of genotype to phenotype (G2P) modeling approaches. We generate and analyze response surfaces, or adaptation landscapes, for yield and yield related traits, emphasizing the organization of the traits in a hierarchy and their development and interactions over time. We use the crop growth model APSIM-wheat with genotype-dependent parameters as a tool to simulate non-linear trait responses over time with complex trait dependencies and apply it to wheat crops in Australia. For biological realism, APSIM parameters were given a genetic basis of 300 QTLs sampled from a gamma distribution whose shape and rate parameters were estimated from real wheat data. In the simulations, the hierarchical organization of the traits and their interactions over time cause G×E for yield even when underlying traits do not show G×E. Insight into how G×E arises during growth and development helps to improve the accuracy of phenotype predictions within and across environments and to optimize trial networks. We produced a tangible simulated adaptation landscape for yield that we first investigated for its biological credibility by statistical models for G×E that incorporate genotypic and environmental covariables. Subsequently, the simulated trait data were used to evaluate statistical genotype-to-phenotype models for multiple traits and environments and to characterize relationships between traits over time and across environments, as a way to identify traits that could be useful to select for specific adaptation. Designed appropriately, these types of simulated landscapes might also serve as a basis to train other, more deep learning methodologies in order to transfer such network models to real-world situations.

## Background

A major objective of breeding programs is to create and identify genotypes that are well adapted to the growing conditions in which the resulting varieties are likely to be grown. This set of conditions is referred to as the target population of environments, TPE (Comstock and Moll, 1963; Cooper and Hammer, 1996; Chenu, 2015). Across the TPE, climate, soil, and management typically change, inducing genotype-specific responses that might lead to heterogeneous genotypic ranking for the target trait (e.g. yield). For example, the Australian grain cropping TPE has been represented by four or five environment types (ETs) that correspond to different water deficit patterns (sorghum: Chapman et al., 2000b; wheat: Chenu et al., 2011; Chenu et al., 2013). These water deficit patterns induce genotype-specific responses that are an expression of genotype-by-environment interaction (G×E).

G×E for yield (i.e. the target trait) is an emerging property of agricultural systems and results from the interplay between secondary traits, organized in a hierarchy that involves the capture and allocation of environmental resources during the growing season (Chapman et al., 2002b; Hammer et al., 2016). Traits underlying yield differ in their contribution to adaptation across environmental conditions; e.g. later flowering might be advantageous for yield in a non-dry environment but can be counterproductive in an environment with late drought (Slafer et al., 2005). Secondary traits can be classified as intermediate traits (e.g. biomass, flowering time, grain number) or as basic traits that correspond to response mechanisms/sensitivities to the environmental conditions (e.g. sensitivity to photoperiod, radiation use efficiency). As the target and intermediate traits involve a large number of processes, they are more prone to G×E. In contrast, basic traits/sensitivities usually show less G×E because they correspond to mechanisms of response to the environment; they are less context-dependent, but they sense and generate the environmental context for more complex traits (Tardieu and Tuberosa, 2010a; Hammer et al., 2016; Chenu et al., 2018). Although genotypic sensitivities to environmental conditions are hard to phenotype, their estimates facilitate predictions along the environmental gradient, provided that the relevant environmental variables are measured (Tardieu and Tuberosa, 2010b; van Eeuwijk et al., 2019). As traits along the trait hierarchy might interact in non-linear ways, G×E for the target trait can be observed, even if its underlying traits do not show G×E (Génard et al., 2017).

To study G×E, breeders traditionally evaluate genotypes in multi-environment trials (METs) consisting of a sample of locations and years that is hoped to represent the TPE. Practical constraints restrict the number of genotype–environment combinations that can be tested in METs, as well as the number and frequency of traits that can be measured. Therefore, it is typically hard to understand from the analysis of MET yield data how G×E has arisen from component traits and the biological interactions between them as well as their responses to the environment. It will be useful to consider the dynamics of the underlying traits (intermediate traits like biomass and basic traits/sensitivities). Such traits will be genetically correlated to yield, making them interesting targets for selection on specific adaptation (Furbank et al., 2019). Intermediate and basic traits are becoming increasingly accessible thanks to high-throughput phenotyping techniques. As additional phenotyping always implies additional costs, simulations might be used for a more detailed characterization of the system dynamics to identify the key adaptive mechanisms in the targeted conditions. Phenotyping a large number of genotypes and environments over time is still an expensive task, and results typically depend on how representative the MET environments were. Simulations offer an opportunity to evaluate strategies to design METs and efficiently allocate resources (structure and size of the trial network, impact of phenotyping additional traits), compare prediction methods (multi-trait, multi-environment genomic prediction, and QTL models), and develop methodologies to assess how additive effects for basic mechanisms influence intermediate and target traits, among others.

Our simulations involved the combination of statistical-genetic models and crop growth models (Chapman, 2008; van Eeuwijk et al., 2019). A number of interesting approaches for crop growth models with genotype-dependent parameters have been proposed during the past decades. For example, Chapman et al. (2002a,2003) and Chapman (2008) simulated a number of sorghum genotypes varying in APSIM parameters. APSIM is an example of a class of widely-used crop growth models that account for characteristics from the crop, weather, soil, and agronomic management and their interactions over time (Wang et al., 2002; Keating et al., 2003; Holzworth et al., 2014). The algorithms in APSIM predict yield as a nonlinear combination of intermediate traits, which are calculated indirectly from environmental conditions and a number of physiological parameters (sensitivities and partitioning coefficients) that are constant across environments. The simulations by Chapman et al. (2002) and Chapman (2008) produced realistic G×E patterns and allowed to connect G×E to explicit water deficit scenarios for a limited set of discrete genotype classes, varying for four physiological traits. A similar approach, implemented at a larger-scale on representative cropping conditions, was applied for wheat by Chenu et al. (2011; 2013), who used a detailed soil and long-term meteorological characterization to assess the frequency of occurrence of different drought patterns along the Australian wheat belt for three genotypes differing in phenology.

The novel aspect of this paper, is that we added an explicit and detailed quantitative genetic basis to simulated APSIM-wheat physiological parameters. In this way, we simulated multiple traits over time and across environments, with a quantitative genetic basis. To make our simulations realistic, APSIM-wheat parameters were genetically regulated by additive QTL effects that follow the same distribution as the ones observed for real phenotypic data for an association panel in Australia (data also used in Bustos-Korts et al., 2016a). Parameter ranges were adjusted to those that have been observed in physiological experiments for wheat. The genotype-specific parameters with known genetic basis were used to simulate daily phenotypes of secondary traits (e.g. biomass, canopy cover, flowering time) for 199 genotypes growing in four Australian locations during 31 years. In this paper, we simulated data that were first explored for the structure of G×E by statistical models that incorporated genotypic and environmental covariables to assess the biological credibility of the simulated data. Subsequently, we used the simulated data to evaluate statistical genotype-to-phenotype models for multiple traits and multiple environments. Finally, we studied relationships between traits over time and across environments, as a way to identify traits that could be useful in selection for specific adaptation.

## Methods

The Methods section is presented in three sub-sections: sub-section *Genotypic*, *Phenotypic*, *and Climatic Data Used to Define Simulation Settings* describes the real data (markers and phenotypes) used to do a genome-wide-association analysis and define the environmental conditions in the simulations; sub-section *Crop Simulations* describes the steps to define the distributions for the simulated QTL allelic effects underlying the APSIM parameters and the simulation settings; sub-section *Genetic*, *Environmental*, *and G×E Analyses of the Simulated Yield Response Surface* describes genetic, environmental, and G×E analyses of the simulated phenotypes.

### Genotypic, Phenotypic, and Climatic Data Used to Define Simulation Settings

#### Genotypic Data

Genotypic data consisted of SNPs for a sample of the target population of wheat genotypes (TPG) for Australia, containing 199 genotypes (Australian Wheat Flowering time Association Mapping panel, AWFAM) that represent the range in flowering time variation that is relevant for Australian wheat. This panel was previously used in research about phenotype prediction in Australian environments (Zheng et al., 2013; Bustos-Korts et al., 2016a). Genotypes were characterized with 3,035 polymorphic SNPs with less than 1% missing data and with a minor allele frequency larger than 0.02 (**Tables S1** and **S2**). SNPs and map are provided in the Supplementary material. Genotypes were characterized for their alleles for photoperiod and vernalization, following the same procedure as described in Zheng et al. (2013). Missing markers were imputed using the *missForest* package in R (Bogard et al., 2014).

To characterize the structure of the AWFAM population, a relationship matrix ***A*** was calculated from the SNPs following Patterson et al., (2006):

(1) $$A=\frac{XX^{\prime }}{n_{m}}$$

In model (1), the elements in the matrix ***A*** are proportional to the genetic covariance among genotypes. ***XX*′** is the product of a matrix of dimensions number of genotypes by number of SNPs (***X***) and its transpose (***X*′**), whose entries are the standardized marker scores, coded 0, 1, or 2, representing the number of copies of the minor allele. *n_m_* is the number of markers.

To study population structure, we inferred the number of subpopulations present in AWFAM from the number of significant principal components, calculated after applying a spectral decomposition to the matrix ***A*** (Patterson et al., 2006). Genotypes were grouped and assigned to subpopulations using a hierarchical clustering procedure applied to the significant principal components, following Odong et al. (2013). The cut-off for the dendrogram was chosen such that the number of subpopulations was equal to the number of significant (p<0.05) principal components plus one. Pearson correlation between each of the APSIM parameters and the kinship principal components were calculated and added as vectors in a biplot depicting the genetic diversity.

#### Phenotypic Data

Phenotypic data consisted of plot observations for yield and heading date of the 199 genotypes belonging to the AWFAM panel observed in eight environments across the Australian wheat belt. A row–column design with two replicates was used. Adjusted means were calculated with the following mixed model:

(2) $$y_{ijl\left(k\right)}=\mu +Rep_{k}+R_{j}+C_{l\left(k\right)}+G_{i}+\varepsilon_{ijl\left(k\right)}$$

In model (2), *y_ijl(k)_* is the phenotype of genotype *i* in replicate *k*, row *j*, and column *l* within replicate *k*, *µ* is the intercept, *Rep_k_* is the fixed effect of replicate *k*, *R_j_* is the random effect of row *j*, *C_l(k)_* is the random effect of column *l* within replicate *k*, *G_i_* is the fixed effect of genotype *i*, and ε*_ijl(k)_* is the vector of spatially correlated residuals modelling the local trend, with distribution ε*_ijl(k)_*∼*N*(0, ***R***). ***R*** represents the Kronecker product of first-order autoregressive processes across rows and columns, and $\sigma_{e}^{2}$ is the residual variance: $R=\sigma_{e}^{2}(AR1⊗AR1)$.

#### Environments

APSIM-wheat simulations were carried out to generate a sample of the TPE defined by four sites (Emerald, Narrabri, Yanco, and Merredin) and 31 years (1983–2013), corresponding to a subset of the environments used in Casadebaig et al. (2016) and Chenu et al. (2013). This subset was chosen to represent common conditions at four contrasting wheat growing areas in the Australian wheat belt. Climate data were sourced from the SILO patched point data set (http://www.longpaddock.qld.gov.au/silo/index.html; Jeffrey et al., 2001) and are summarized in **Table 1** and **Figure S1**.

**Table 1:** Characteristics of the locations, soils, and management regimes representing the target population of environments considering the period 1983–2013.

Variable	Emerald	Merredin	Narrabri	Yanco
Latitude (degree)	−23.53	−31.50	−30.32	−34.61
Longitude (degree)	148.16	118.22	149.78	146.42
Rainfall pattern	Summer dominant	Winter dominant	Summer dominant	Evenly distributed
Annual rainfall (mm)	585	313	644	406
Seasonal rainfall (mm)	89	181	202	193
Daily mean temperature (^o^C)	17.5	12.9	13.5	11.9
Daily mean radiation (MJ m^−2^)	16.7	14.4	15.0	13.6
Soil type	Black vertosol	Shallow loamy duplex	Gray vertosol	Brown sodosol
PAWC (mm)	133.5	101.1	217.5	190.8
Sowing date	15 May	15 May	15 May	15 May
Sowing PAW (mm)	132	39	175	99
Soil nitrogen at sowing (kg ha^−1^)	30	30	30	50
Applied nitrogen (kg ha^−1^)	50/0/0	20/20/30	130/0/0	40/40/40

Plant available water capacity (PAWC) is indicated for each soil, as well as the level of plant available water (PAW) at sowing which was used in the simulations, following (Casadebaig et al., 2016). Applied nitrogen doses are indicated by a/b/c, with the fertilization applied at sowing (a), at the stage of end of tillering (b), and at the stage mid-stem elongation (c). Seasonal data considered the growing period between 15 of May and the maturity date averaged across genotypes.

### Crop Simulations

**Figure 1:**
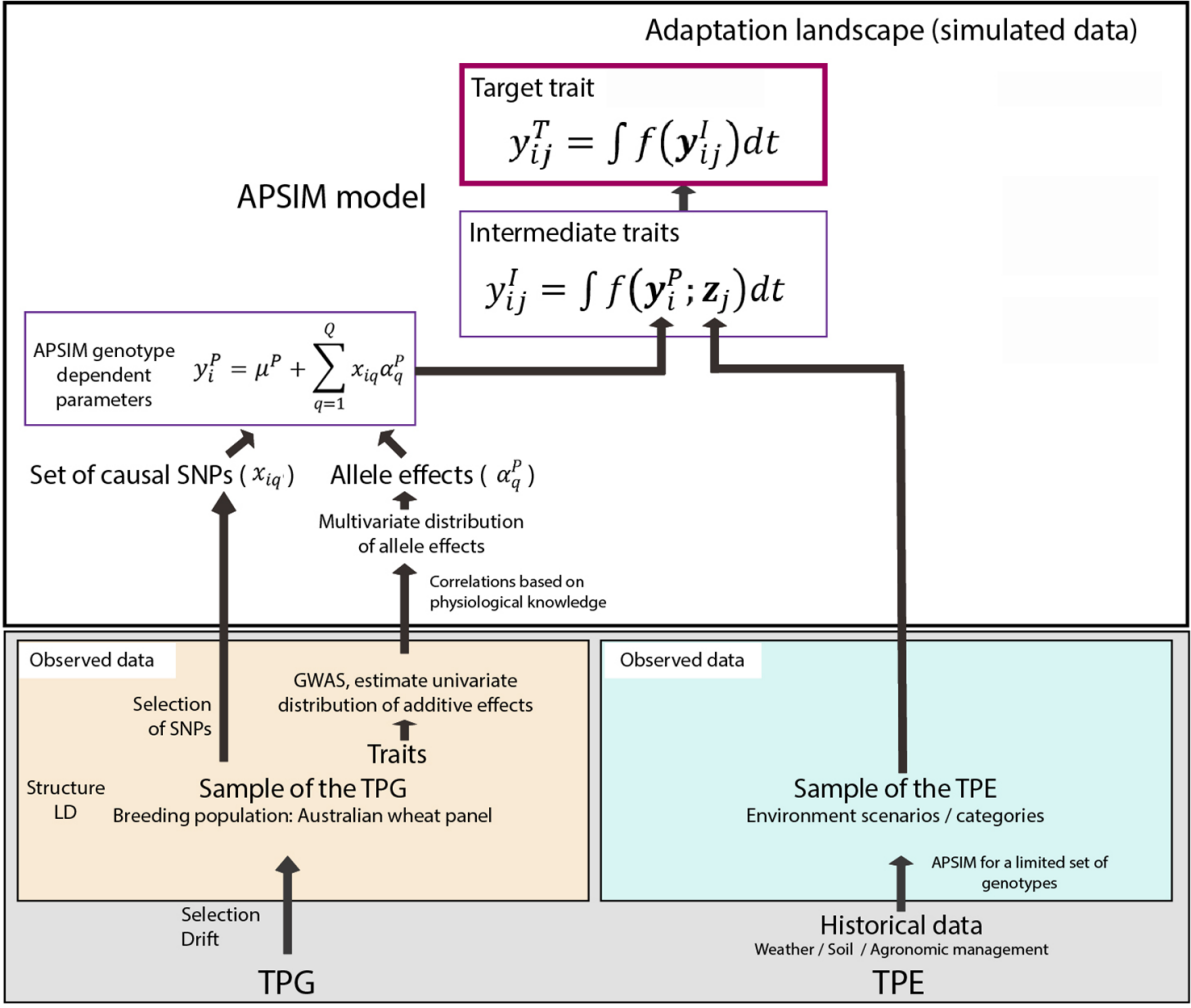
Steps to generate the adaptation landscape. Bottom left; an Australian wheat panel is defined as a sample of the target population of genotypes (TPG). For this set of wheat lines, the genotypes were characterized by SNP markers, phenotypic data have been collected in field trials. The phenotypic and genetic data were used in univariate GWAS analyses to estimate empirical distributions for the additive effects of QTLs underlying these phenotypes. Physiological knowledge on trait correlations was used to define genetic correlations between APSIM parameters $\left(y_{i}^{P}\right)$. These correlations are included in a multi-variate description of the QTLs underlying APSIM parameters. From this distribution, genotype specific APSIM parameters $\left(y_{i}^{P}\right)$ are generated and assigned to a subset of SNPs. Bottom right; 31 years of historical environmental data at four sites were used to define the target population of environments (TPE) and identify contrasting environment scenarios (water deficit patterns). Top panel; the environmental data of the selected scenarios and the genotype-dependent APSIM parameters are used to generate intermediate traits over time $\left(y_{ij}^{I}\right)$ using APSIM. The target trait $\left(y_{ij}^{T}\right)$ is modeled as a function of intermediate traits.

Phenotypic data was simulated for the 199 studied genotypes characterized by 12 genotype-specific at the four studied sites over 1983–2013 (**Figure 1**) for standard management practices with the APSIM model, version 7.7 (Holzworth et al., 2014). Sub-section *Genotype-Specific Parameters and Their Genetic Basis* describes the generation of genotype specific values for 12 APSIM parameters, and sub-section APSIM Crop Simulations describes the settings that were used when running the APSIM model for a sample of the TPG and TPE.

#### Genotype-Specific Parameters and Their Genetic Basis

**Figure 2:**
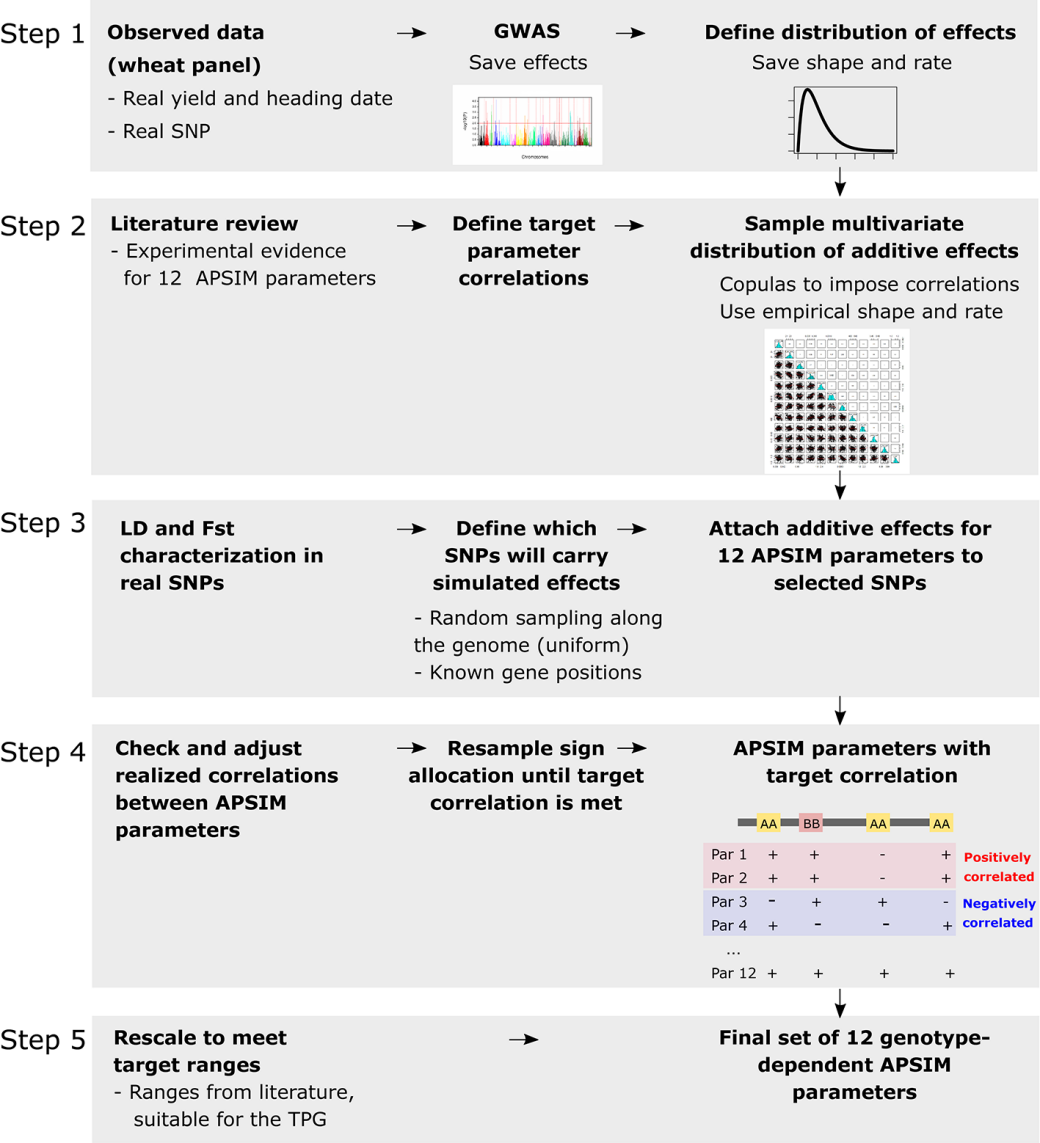
Steps to generate the genotype-dependent parameters, additive effects sampled with copulas from a marginal distribution that follows the same shape and rate than the ones of real wheat data. Steps were as follows: (1) define the distribution of the underlying additive effects by fitting empirical distributions of additive effects estimated from a genome-wide association scan (GWAS) applied to wheat heading date and yield in Australia, (2) sample the additive effects from gamma marginal distributions using copulas, (3) attach the sampled additive effects to 300 SNPs randomly sampled and in low linkage disequilibrium with each other. Alleles for heading date were attached to known flowering time genes. The fixation index (Fst) was calculated for each of the 300 SNPs to assess its potential confounding with population structure. (4) For each APSIM parameter, define which will be the trait-increasing allele, so that target parameter correlations are met, and (5) rescale parameters to meet the range that is biologically relevant for this target population of genotypes (TPG).

Genotype-specific values were generated for 12 APSIM parameters, regulating phenology, capture of environmental resources, resource use efficiency, and biomass partitioning (**Figures 1** and **2**). Parameter selection was based on their impact on grain yield, as shown by a global sensitivity analysis (Casadebaig et al., 2016) and by physiological studies (e.g. Manschadi et al., 2006; Acreche et al., 2009; Schoppach and Sadok, 2013). The set of 12 parameters was generated in the following steps (**Figure 2**): (1) define the distribution of the underlying QTL additive effects, (2) sample the QTL additive effects from Gamma marginal distributions using copulas (Nelsen, 2013), (3) attach the sampled additive effects to 300 SNPs along the genome, (4) for each APSIM parameter, define which will be the trait-increasing allele, so that target parameter correlations are met, and (5) rescale parameters to meet the range that is biologically relevant for this TPG.

The first step (**Figure 2**) consisted in defining the distribution of the underlying additive QTL effects. These QTL effects were estimated from the application of a single locus GWAS on heading date and yield of the AWFAM panel, observed in the Australian wheat belt (adjusted means obtained in section *Genotypic Data*).

(3) $$y_{i}=\mu +x_{ik}\alpha_{k}+G_{i}+\varepsilon_{i}$$

In model (3), *y_i_* stands for the phenotype of genotype *i*, *µ* is the intercept, *x_ik_* is a vector that represents information of genotype *i* at marker *k* (0, 1, or 2 for the number of minor alleles), and *α_k_* is the additive QTL effect (fixed) for marker *k*. *G_i_* represents a polygenic effect for genotype *i*, with distribution $G_{i}\sim N\left(0,A\sigma_{g}^{2}\right)$. ***A*** is the chromosome-specific kinship matrix calculated from the molecular marker information as in Rincent et al. (2014). ϵ*_i_* is the residual $\left(\varepsilon_{i}∼N\left(0,\sigma_{e}^{2}\right)\right)$. The empirically estimated additive effects (*α_k_*) were used to define the shape and rate of the marginal gamma distributions to be followed by the simulated data (step 1 in **Figure 2**). The estimation of the parameters for these distributions was done by maximum-likelihood using the DISTRIBUTION directive in Genstat 18 (VSN-International, 2016). As the distribution parameters slightly differed between environments, we used the median of the Gamma shape and rate across environments (Step 1 in **Figure 2**, **Table 2**).

**Table 2:** Genotype-specific parameters from APSIM that were used in this study, with the lower and upper limits for the simulated population, and the default value of the parameter in the released version of APSIM.

Parameter	APSIM name	Units	Default APSIM	low_lim	high_lim	References
Fraction of leaves senescing per main stem node	fr_lf_sen_rate	[]	0.035	0.028	0.042	(Christopher et al., 2016)
Number of grains per gram of stem at flowering	grains_per_gram_stem	grains $g_{stem}^{-1}$	25	20	25	(Dodig et al., 2012)
Lower limit for water uptake	ll_modifier	[]	1	0.9	1.1	(Manschadi et al., 2006)
Maximum grain size	max_grain_size	mg	0.041	0.03	0.06	(Groos et al., 2003)
Sensitivity to photoperiod	photop_sens	[]	2	1.5	3	(Zheng et al., 2013)
Potential grain filling rate	potential_grain_filling_rate	g grain^−1^ day^−1^	0.0025	0.0016	0.0026	(Wang et al., 2009)
Transpiration efficiency coefficient	transp_eff_cf	[]	0.006	0.0045	0.0065	(Fletcher and Chenu, 2015; Fletcher et al., 2018)
Thermal time required to reach floral initiation	tt_floral_initiation	^o^C d	555	455	555	Within the range of (Zheng et al., 2013)
Sensitivity to vernalization	vern_sens	[]	1.5	1.5	2.5	(Zheng et al., 2013)
Canopy extinction coefficient	y_extinct_coef	[]	0.45	0.4	0.6	(Isidro et al., 2012)
Biomass partitioning to leaf	y_frac_leaf	[]	0.6	0.55	0.65	(Álvaro et al., 2008)
Radiation use efficiency	y_rue	g MJ^−1^	1.24	1.01	1.4	(Acreche et al., 2009)

For all traits, the marginal Gamma distribution had the following parameters; shape (determining the skewness) k = 1.299 and a rate (inverse scale parameter) b = 13.6, except for photop_sens, tt_floral_initiation and vern_sens, for which k = 0.700 and b = 13.6. When units are indicated with [], it means that the parameter is dimensionless.

In step two (**Figure 2**), we defined the additive effects producing the APSIM parameters. We used physiological knowledge reported in the literature to specify correlations between APSIM parameters. The genetic basis of the simulated APSIM parameters was established by 300 SNPs, assumed to have additive effects; no epistasis was modeled for any of these parameters. The SNP effects were sampled from the gamma distribution with the shape and rate parameters estimated from the GWAS analysis of heading date and yield observed for real phenotypic data. The univariate distributions for physiological parameters were turned into a multi-variate distribution by considering the physiological evidence for correlations between some of the APSIM parameters. Most of the parameters were assumed to be uncorrelated, except for the transpiration efficiency coefficient and radiation use efficiency (r = -0.40), the number of grains per gram of stem at flowering and maximum grain size (r = -0.50) and maximum grain size and potential grain filling rate (r = +0.45). These correlations were set to match physiological constraints that have been observed in real experiments (Slafer and Savin, 1994; Monneveux et al., 2006; Sadras and Lawson, 2011; Bustos et al., 2013).

To impose parameter correlations, 300 additive SNP effects were sampled with copulas (step 2, **Figure 2**), implemented in the R package *copula* (Maechler, 2017). Briefly, copulas describe the dependence between random variables, each of them with a separate marginal Gamma distribution for the additive effects (Nelsen, 2013). Copulas allow the creation of correlations between the additive effects. Since the Gamma distribution always takes positive values, 50% of the additive effects for each parameter were randomly assigned a negative sign (Pérez-Enciso et al., 2017). In this way, the major and the minor allele had 50% probability of increasing the trait (on average, across the 300 SNPs). Sign allocation was done independently for each parameter, but an exception was made for the following pairs of correlated parameters: (a) transpiration efficiency coefficient and radiation use efficiency, (b) grains per gram of stem and maximum grain size, and (c) maximum grain size and potential grain filling rate. In these three exceptional cases, the sign was assigned jointly for a proportion of the additive effects, instead of independently, as for the other parameters (step 4, **Figure 2**). If target parameter correlation was positive, a negative sign was assigned jointly to 60% of the most common allele of randomly sampled SNPs (i.e. the same allele would increase the value of both parameters), whereas for the other 40% of the SNPs, the trait-increasing allele was assigned for each parameter to either of the alleles. From a range of percentages, the value of 60% was chosen because it allowed best to achieve the target correlations between APSIM parameters. If the target parameter correlation was negative (in the case of variable pair: grains per gram of stem and maximum grain size), opposite signs were assigned randomly to a 60% of the additive effects. This means that the least common allele of 60% of the loci would be assigned a negative sign for one of the correlated parameters and a positive sign for the other correlated parameter. Therefore, the correlation between each pair of APSIM physiological parameters was determined via the correlation between the absolute value of the QTL additive effects imposed by the copula, and by the proportion of loci for which the same allele is increasing both APSIM parameters.

In step three (**Figure 2**), we defined QTL locations. Positions of the 300 SNPs with additive effects were determined by sampling from a uniform distribution. Because of the random nature of the sampling process, some SNPs were more related to population structure than others. For that reason, SNPs with additive effects differed in their fixation index values (Fst, Wright, 1949). Additive effects sampled in the second step above described were assigned at random to the 300 SNPs, except for the largest additive effects regulating photoperiod sensitivity (photo_sens) and vernalization requirements (vern_sens). In this case, the largest additive effects were assigned to SNPs at the position of nine known flowering time genes/QTLs, which showed moderate to large Fst (between 0.11 and 0.72 with a median of 0.5). The consequence of this decision was that photo_sens and vern_sens were more likely to be correlated to population structure, making it more difficult to detect QTLs using GWAS methods. We used the 300 SNPs and their additive effects to generate genotype-specific APSIM parameter values for each genotype, Equation (4), step 3 in **Figure 2**. These genotype-specific parameters are constant across environments.

(4) $$y_{i}^{p}=\mu^{p}+\sum_{q=1}^{Q}x_{iq}\alpha_{q}^{p}$$

In model 4, $y_{i}^{p}$ is the vector with genotypic values that were simulated for APSIM parameter *p* (*p* = 1…12); *µ^p^* is the mean of the range for the simulated APSIM parameter *p; x_iq_* is an indicator variable with values −1, 0, and 1 that represents information of genotype *i* at marker *q*; $\alpha_{q}^{p}$ is the additive QTL effect for marker/QTL *q* and parameter *p*. In step 4, we checked the realized correlations between APSIM parameters. If the correlation was very different from the target, we rejected that sample and repeated the sampling for sign allocation until the target correlation was achieved (**Figure S2**). Samples were rejected when pairs of parameters that should have a low correlation had a correlation larger than 0.4, and when pairs of parameters that were intended to be correlated departed more than ±0.2 of the target correlation. If a sample was rejected, we repeated from step 3 onwards (sign allocation) until the realized correlations were within the target range. In step 5 (**Figure 2**), we rescaled the resulting APSIM parameters, to meet the ranges shown in **Table 2**.

#### APSIM Crop Simulations

Crop simulations were conducted with the APSIM-wheat model for the 199 studied genotypes characterized by the 12 physiological parameters at Emerald, Narrabri, Yanco, and Merredin using historical climatic data from 1983–2013 and soil characteristics as described in **Table 1**, **Figure S1**, and section Environments. Sowing settings corresponded to control conditions from Casadebaig et al. (2016), chosen to mimic local farming practices. Sowing date was set at the 15th of May for all sites, and the soil conditions were reset each season, i.e. we did not simulate a continuous wheat cropping system, but treated the seasons as independent environment samples, assuming that the genotypes were sown into representative soil conditions. Further details on the sowing and management parameters can be found in **Table 1** and in Casadebaig et al. (2016).

The 12 simulated parameters showing variation for the 199 genotypes were used to generate intermediate traits using APSIM, and expressed as equation (5).

(5) $$y_{ij}^{I}=\int f\left(y_{i}^{P};z_{j}\right)dt$$

where $y_{ij}^{I}$ is an intermediate trait for genotype *i* in environment *j*, which is modeled as a function of multiple APSIM parameters/inputs, $y_{i}^{P}$, (12 of them that are genotype-specific) and multiple environmental inputs, *z_j_*, integrated over time (**Figure 1**). Description and ranges of the genotype-dependent APSIM parameters can be found in **Table 2**. For a more detailed description of APSIM see the user manual (http://www.apsim.info/; Zheng et al., 2015). The function *f* embodies the algorithms that transform APSIM parameters and environmental inputs into intermediate phenotypes, $y_{ij}^{I}$ (e.g. biomass, grain number, grain weight). In APSIM, the target trait (yield) for genotype *i* in environment $j\left(y_{ij}^{T}\right)$ is modeled as a function of intermediate traits and the environment over time, following equation (6);

(6) $$y_{ij}^{T}=\int f\left(y_{ij}^{I}\right)dt$$

The APSIM simulations sampled part of the Australian wheat TPE comprising environments with standard management at four locations between 1983–2013. The saved output of the APSIM simulation consisted of phenology, dry biomass and yield for each genotype, and indices for environmental characterization (cumulative thermal time, soil water supply, and soil water demand) for each day during the whole growing season.

### Genetic, Environmental, and G×E Analyses of the Simulated Yield Response Surface

In this section, we describe statistical models that we applied to analyze the simulated data. The simulation data were analyzed for three distinct purposes. Firstly, we need to verify whether the genotype-by-environment data as simulated by APSIM have a realistic structure, i.e., whether the G×E patterns that we detect in the simulated data correspond with the patterns that occur in empirical MET data. If the simulated data have the characteristics of real-world experiments, they provide an opportunity to investigate the performance of purely statistical models for yield prediction. A third type of analysis of our simulated data consist of examining the correlations between traits over time and across environments. In a companion paper, we discuss whether statistical prediction models for yield can be improved by using additional information on the dynamics of secondary traits.

#### G×E Analyses

For our simulations to be useful to answer questions related to genotype adaptation across the Australian TPE, the size and nature of G×E need to resemble that of experimental data. Therefore, APSIM yield and biomass at harvest simulated for the 199 genotypes, 31 years, and four locations were used to investigate and describe the G×E patterns in the sample of the TPG and TPE that we simulated. A common way of characterizing G×E in empirical breeding data is by fitting an Additive Main Effects and Multiplicative Interaction (AMMI) model followed by an inspection of genotypic and environmental scores (Gauch and Zobel, 1997; Gauch, 2013; van Eeuwijk et al., 2016).

(7) $$y_{ij}=\mu +E_{j}+G_{i}+\sum_{m=1}^{M}b_{im}z_{jm}+\varepsilon_{ij}$$

In model (7), *y_ij_* represents the APSIM output for yield without error of the ith genotype in the jth environment, *µ* stands for the intercept, *G_i_* is the fixed effect of the *i*^th^ genotype and *E_j_* is the fixed effect of the jth environment. The interaction in an AMMI model is described by *M* multiplicative terms that consist of products of the genotypic sensitivity *b_im_* (genotypic score) and an environmental score *z_jm_*. Finally, ϵ*_ij_* is a residual term that contains the part of the two-way analysis of variance interaction that is not explained by the AMMI interaction terms. Genotypic and environmental scores allow visualizing the G×E interaction patterns in the form of biplots. We used an AMMI-2 biplot (scatter plot of the first two multiplicative terms) to assess whether the G×E patterns correspond with the water-deficit patterns. Phenotypic correlations were assessed by the angle between the environmental vectors; if the angle is small, those environments can be interpreted as inducing a similar phenotypic response (Kempton, 1984; Malosetti et al., 2013; van Eeuwijk et al., 2016).

A complementary statistical analysis of G×E quantifies the contributions of genotype-by-location, genotype-by-year, and genotype-by-location-by-year interaction variances (Atlin et al., 2011; van Eeuwijk et al., 2016). A three-way mixed model is fitted with the factors genotype, location, and year.

(8) $$y_{ilk}=\mu +L_{l}+Y_{k}+LY_{lk}+G_{i}+GL_{il}+GY_{ik}+GLY_{ilk}$$

In model (8), *y_ilk_* is the phenotype (APSIM output for yield or biomass without error) for genotype *i* in location *j* and year *k* and *µ* stands for the intercept. The term *E_j_* in model (7) was decomposed in fixed terms for locations (*L_l_*), years (*Y_k_*) and the location by year interaction (*LY_lk_*). *G_i_* is the random effect of genotype *i*, *GL_il_* is the random interaction between genotype *i* and location *j*, *GY_ik_* is the random interaction between genotype *i* and year *k* and *GLY_ijk_* is a random term that contains the residual G×E. No extra error term was added to the model because as APSIM is fully deterministic so that the *GLY_ijk_* contains in principle residual G×E.

To quantify the importance of environmental classifications for G×E variation, we define model (9) (details about environment classification are described in the following section).

(9) $$y_{ijt}=\mu +T_{t}+E{\left(T\right)}_{j\left(t\right)}+G_{i}+GT_{it}+GE{\left(T\right)}_{ij\left(t\right)}$$

In this model, *T_t_* is a fixed environment type effect, *E(T)_j(t)_* is the fixed effect of environments (trials) within environment types, *GT_it_* is the random genotype by environment type interaction and *GE(T)_ij(t)_* is residual G×E due to trials within ET variation.

To evaluate the dynamics of G×E over time, we selected three single environments (trials) that showed contrasting genotypic responses (by looking at the AMMI biplot.). In those selected environments, we fitted on a daily basis a simple two-way mixed model (10) to quantify the contribution of G×E to the total phenotypic variance for biomass for each day during the growing season.

(10) $$y_{ij}=\mu +E_{j}+G_{i}+GE_{ij}$$

Here, *y_ij_* is the APSIM output for biomass without error for genotype *i* in environment *j*, *E_j_* is the fixed environment effect, *G_i_* is the random effect of genotype *i* and *GE_ij_* is the random G×E. For each environment, we quantified the autocorrelation for biomass over time. The autocorrelation was calculated as the Pearson correlation coefficient between biomass lagged by 5, 10, and 15 days [biomass at day *t* and day (t − 5, 10, or 15)].

#### Environment Classification

In addition to implicit environment characterizations based on phenotype data, as can be obtained from AMMI analyses, we also grouped trials using explicit environmental information about the dynamics of water deficit patterns during the growing season. To calculate the water deficit patterns, we ran APSIM for a “standard” genotype that had the average population value for each parameter (*µ^p^*). We calculated the water supply/demand ratio to provide an explicit representation of the water availability in the soil, as perceived daily by the crop (Chapman et al., 2000b; Chenu et al., 2011; Chenu et al., 2013). The water supply/demand ratio indicates the degree to which the soil water extractable by the roots (water supply; associated with root depth and root/soil water conductance) is able to match the potential transpiration (water demand). The water demand (mm) corresponds to the amount of water the crop would have transpired in the absence of soil water constraints and is estimated on a daily basis from the potential radiation-driven crop growth on that day (g m^–2^), the genotype transpiration efficiency (g mm^-1^ water) and the atmospheric saturation vapor pressure deficit (kPa). Water supply–demand ratio for each environment was centered around flowering and averaged over 100°Cd from emergence to 450°Cd after flowering. Environments were classified into four ETs, applying hierarchical clustering to the dynamics of water supply–demand ratios.

#### Trait Correlations Over Time

To study the impact of APSIM parameters on the dynamics of intermediate traits and final yield, we calculated Pearson correlations between simulated APSIM parameters and the simulated daily values for intermediate traits and between simulated APSIM parameters and simulated final yield (APSIM output without error). These correlations were calculated for three of the environments that induce the most contrasting genotypic responses, as selected from an AMMI biplot (section *Genetic*, *Environmental and G×E Analyses of the Simulated Yield Response Surface*).

#### QTL Effects Over Time

To describe G×E at the genetic level, we did a genome-wide association analysis using a single locus GWAS model as described above in Equation 3. The model was fitted to the APSIM output for daily biomass, in each of the three most contrasting environments. The additive QTL effects were expressed as a percentage of the daily biomass mean.

#### Genomic Prediction

Various scenarios need to be distinguished for multi-environment genomic prediction, depending on whether genotypes, environments, or combinations thereof, are to be predicted (Bustos-Korts et al., 2016b; Malosetti et al., 2016; Ly et al., 2017). In this paper, we focus on the prediction of unobserved environments, assuming that all genotypes have been observed/phenotyped in the training set, i.e. each training set encompassed the full set of 199 genotypes. As G×E in the Australian wheat belt is mostly driven by water deficit patterns, we constructed 20 training sets, each of them consisting of 16 simulated trials that were drawn in a stratified random fashion from the four ETs, i.e. each training set consisted of 4 × 4 trials. For each of the 20 training sets, the remaining 108 simulated trials between 1983–2013 were used for validation.

The simulated APSIM yield genotypic values do not contain error because APSIM is a fully deterministic model. Therefore, phenotypic differences in the same environment can be interpreted as genetic. To evaluate the genomic prediction models, we added an error to the APSIM output for yield and the physiological APSIM parameters (for the analyses described in sections *G×E Analyses, Environment Classification, and QTL Effects Over Time*, we used the APSIM yield and parameters without error).

(11) $$H^{2}=\frac{\sigma_{g}^{2}}{\sigma_{g}^{2}+\sigma_{e}^{2}}$$

In equation (11), the genotypic variance at a given environment $\left(\sigma_{g}^{2}\right)$ was calculated as the variance of APSIM yield for that environment. The experimental error $\left(\sigma_{e}^{2}\right)$ was sampled independently for each environment and added to the APSIM outputs for yield. For yield, heritability was set to 0.50. APSIM parameters used for the genomic prediction models had a heritability of 0.70. Four types of genomic prediction models were evaluated:

##### Genomic Prediction Models Using an ET Mean (ETmean)

This model uses an environmental classification based on four ETs (see section *Environment Classification*) to structure the variance–covariance matrix across environments. As the four relevant ETs are represented in the training set because of stratified sampling, unobserved environments (trials) can be predicted from the means of the ET to which that trial belongs to, provided that the ET in the validation environment is known:

(12) $$y_{ij\left(t\right)}=\mu +T_{t}+E{\left(T\right)}_{j\left(t\right)}+G_{it}+\varepsilon_{ij\left(t\right)}$$

In model (12), *y_ij(t)_* is the yield of genotype *i* in environment trial (*j*) belonging to environment type *t* (APSIM output for yield with error to achieve a H^2^ of 0.5), *T_t_* is the fixed effect of each environment type, *E(T)_j(t)_* is the fixed effect of environments (trials) within environment type, *G_it_* is the random effect for genotype *i* in environment type *t*, following *G_it_∼MVN*(0, **Σ**) where **Σ** is a covariance matrix. The variance–covariance matrix **Σ** is modeled as $\sum =\sum_{G}⊗\sum_{E}$, where **Σ***_G_* is the genotypic kinship matrix ***A***, calculated in Equation (7) and **Σ***_E_* is following an unstructured model between the environment types (each environment type has a unique variance and each pair of environment types has a unique correlation). ε*_ij(t)_∼MVN*(0,**R**), where ***R*** is a diagonal structure, allowing for environment type-specific residuals. Predictions for environments that are not in the training set are formed from the means of the environment type to which they belong: ${\hat{y}}_{ij\left(t\right)}=\hat{\mu }+{\hat{T}}_{t}+{\hat{G}}_{it}$.

##### Genomic Prediction Models Based on the Average Product of Principal Components Extracted From the Genotypic and Environmental Kinship Matrices (MeanPC)

In this approach, we used a mixed model including genotypic and environmental covariables to model the variance–covariance matrix between individual trials (year–location combinations), following (Jarquín et al., 2013; Malosetti et al., 2016). The environmental kinship corresponded to the Euclidean distances calculated from standardized environmental covariables calculated for different periods. Environmental covariables consisted of summaries of temperature, solar radiation, water supply–demand ratio, and photoperiod, calculated for each environment during four periods during the growing season (**Table 3**). For computational reasons, we replaced the conventional Kronecker product between the genotypic and environmental kinship in the random part of the mixed model by a simpler fixed analogue based on the cross-products of the principal components of the genotypic kinship and the environmental kinship matrices. These cross-products considered three principal components for the environmental kinship and four principal components for the genotypic kinship (the numbers were defined by testing the significance, following Patterson et al., 2006). After calculating all possible cross-products, the resulting 12 product vectors were averaged to obtain a single vector with one value for each genotype–environment combination, denoted by $x_{ij}^{*}$. The average cross product vector values $x_{ij}^{*}$ were included as fixed effect in the following model:

(13) $$y_{ij}=\mu +E_{j}+\beta x_{ij}^{*}+\varepsilon_{ij}$$

**Table 3:** Environmental covariables calculated using the phenology of the population mean.

Abbreviation	Description
tmean	Mean of the daily average temperatures (°C) above 0
radmean	Sum of the daily radiation (J cm^−2^) (Monteith, 1972)
radtemp	Ratio radmean over tmean (Fischer, 1985)
sd	Mean water supply–demand ratio (output from APSIM)
ct	Sum of the daily minimal temperatures <−4°C
ft	Number of days when the daily minimal temperature is ≤0°C
photo_max	Maximum day length
photo_mean	Mean day length

All variables were calculated between sowing to the start of stem elongation (“P1”), the start of stem elongation to heading (“P2”), heading to 10 days after flowering (“P3”), 10 days after flowering to maturity (“P4”), and for the whole growing season (“whole”).

where *β* represents the sensitivity of yield to changes in the genotypic and environmental distances, as represented by $x_{ij}^{*}$.

##### Genomic Prediction Models Based on the Full Set of Products of Principal Components Extracted From the Genotypic and Environmental Kinship Matrices (IndivPC)

Model (13) uses a single slope to characterize the sensitivity of yield to changes in genotypic and environmental distances. Here, we create a more versatile model by allowing each of the 12 vectors to have a separate slope:

(14) $$y_{ij}=\mu +E_{j}+\sum_{p=1}^{P}\gamma_{p}x_{p,ij}^{\#}+\varepsilon_{ij}$$

In model (14), $\sum_{p=1}^{P}\gamma_{p}x_{p,ij}^{\#}$ represents the *p* = 1…12 vectors corresponding to the cross-products of the three environmental kinship principal components and the four genotypic kinship principal components (see previous paragraph). Each vector is allowed to have a separate slope, represented by *γ_p_*.

##### Prediction Models Based on Factorial Regression With APSIM Parameters (FReg)

To consider a more explicit approach to model G×E, we modeled the sensitivity of APSIM parameters to environmental covariables in a factorial regression model.

(15) $$y_{ij}=\mu +E_{j}+\kappa y_{i}^{p}z_{j}^{*}+\varepsilon_{ij}$$

In model (15), $y_{i}^{p}$ represents a genotype-specific covariable (APSIM parameter with a H^2^ of 0.7) and $z_{j}^{*}$ is an environmental covariable belonging to the set of covariables described in **Table 3**. The proportionality constant κ represents the sensitivity to the product of APSIM parameter and environmental covariable.

We did a forward selection, evaluating all the combinations of APSIM parameters and environmental covariables, also considering the squares of the environmental covariables, sequentially adding the most significant covariable combination in each evaluation round. In the forward selection procedure, the environmental covariables and their squares were added simultaneously. The squares of covariables were dropped if they were not significant, before continuing to evaluate a next covariable and its square. The prediction model with combinations of APSIM parameters, subscript *r*, and environmental covariables, subscript *s*, is then:

(16) $$y_{ij}=\mu_{j}+E_{j}+\sum_{r,s}\kappa_{r,s}y_{ir}^{p}z_{js}^{*}+\varepsilon_{ij}$$

##### Prediction Accuracy

Prediction accuracy was calculated as the Pearson correlation coefficient between the APSIM phenotypes (genotypic value) and the predicted phenotypes (Meuwissen et al., 2001). To comply with the normality assumption, correlation means and standard errors across 30 training set realizations were calculated on a transformed scale using Fischer’s z transformation, $z=\frac{1}{2}\;\left(ln\left(\frac{1+r}{1-r}\right)\right)$. Means and the confidence interval lower and upper bound were back transformed using $r=\frac{\exp\left(2z\right)+1}{\exp\left(2z\right)-1}$ before reporting them.

## Results

In this paper, we simulated the dynamical behavior of a set of secondary traits across multiple environments (trials) as well as values for the primary or target trait, yield, with realistic G×E patterns. The most novel aspect of our paper is that we used explicit distributions for the additive effects included in a multi-locus QTL model regulating 12 of the APSIM parameters. We used the simulated data to evaluate statistical genotype-to-phenotype models that improve the prediction of target phenotypes across environments and to characterize relationships between traits over time and across environments, as a way to identify traits contributing to specific adaptation.

### Exploring the Structure of (Simulated) G×E by Using Models Incorporating Genotypic and Environmental Covariables

We begin the exploration of our simulated data by checking their biological credibility. The joint distribution of simulated secondary and primary traits over time should follow biological principles and possess a time-dependent mean, and a variance-covariance structure that matches that of empirically observed MET data (**Figures S3**–**S5**). When successful, data simulated by a crop growth model with genotype-specific parameters provide a realistic and flexible representation of the empirical adaptation landscape for multiple genotypes. We evaluated to which extent basic traits (APSIM parameters) correlated to population structure, and investigated the relationship between an environmental grouping following from a water-stress index and the values for a set of pedo-climatic characteristics. Further, we looked at grouping of genotypes on the basis of yield and biomass performance across environments.

#### Population Structure

**Figure 3:**
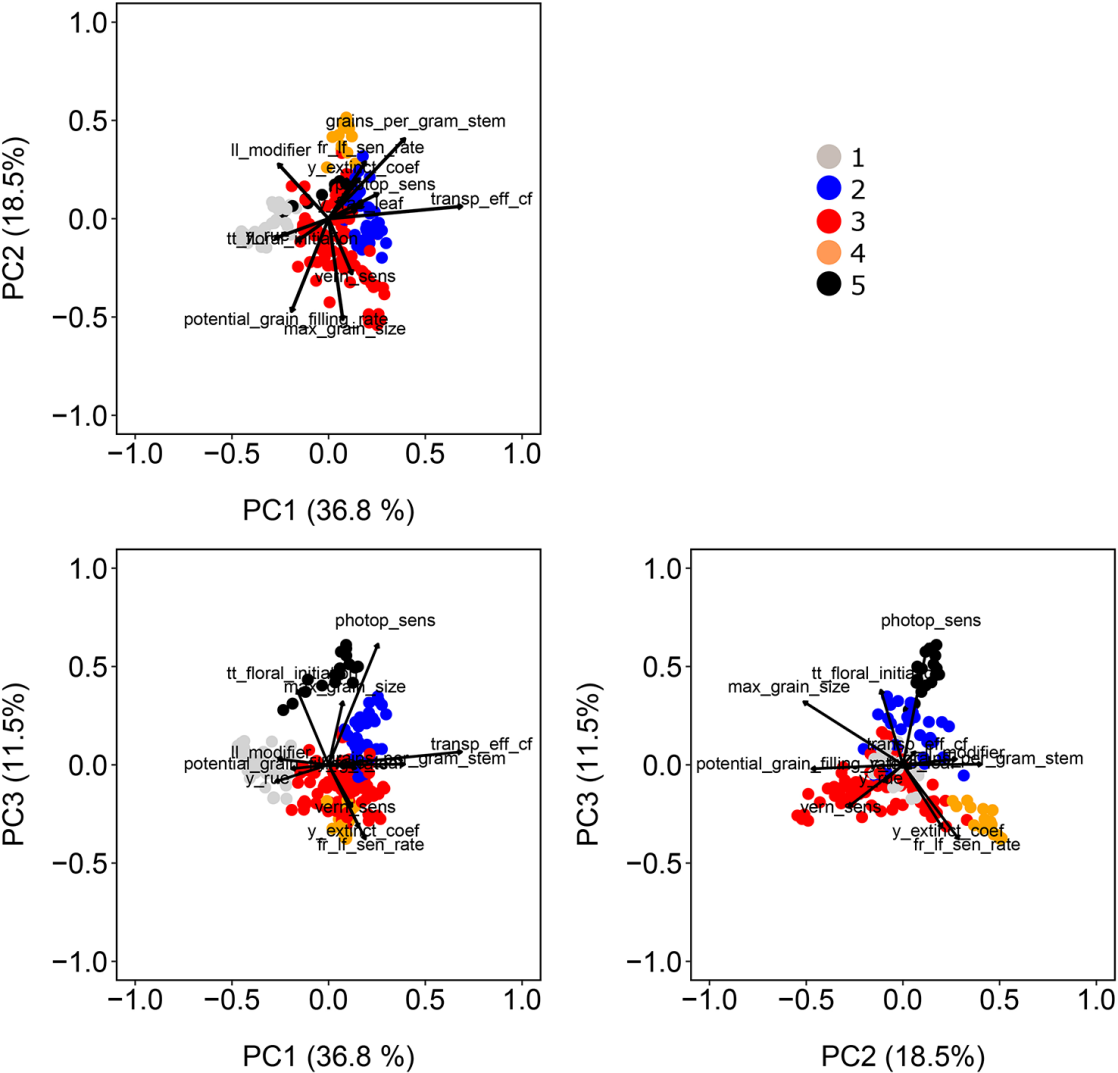
Population structure as revealed by principal components extracted from the matrix of marker scores. Color symbols indicate the genotype assignment to one of the five sub-populations. Directions of greatest change for a set of physiological parameters have been projected on the biplots to help in interpretation. The length of the physiological parameter representations is proportional to the amount of variation explained by the kinship principal components.

For our wheat simulations, we used the AWFAM panel that represents the flowering time variation that is relevant to the Australian TPE, albeit with a greater genomic variation than any single breeding program might utilize for either Australia or a specific region in the wheatbelt. Hence we might assume that the results here are more similar to what might be seen in yield trials of germplasm from earlier stages of breeding program cycle, rather than the results one might find during near-commercial variety testing. Nonetheless, this same approach is applicable to other collections of genotypes. Spectral decomposition of the kinship matrix suggested the presence of five subpopulations (**Figure 3**). These subpopulations coincided to some extent with the frequency of photoperiod and vernalization alleles in the real SNP data of the AWFAM panel. The first eigenvector represented the contrast between subpopulation 1, with mostly photoperiod-insensitive alleles, spring alleles for Vrn-A1, and winter alleles for Vrn-B1, and subpopulation 2 with photoperiod insensitive alleles and a high frequency of winter alleles for Vrn-A1. Along the second eigenvector, subpopulation 3 had mostly photoperiod-sensitive alleles and spring alleles for Vrn-A1 and Vrn-B1. Therefore, the simulated vern_sens had a positive correlation with the first eigenvector and the simulated photop_sens coincided with the direction of the second eigenvector. Other simulated APSIM parameters also were moderately correlated with population structure; i.e. correlations with PC1 ranged from −0.46 (ll_modifier) to +0.31 (vern_sens), correlations with PC2 ranged from −0.27 (y_rue) to +0.69 (transp_eff_cf) and correlations with PC3 ranged from −0.51 (max_grain_size) to +0.40 (grains_per_gram_stem). The intermediate size of the correlation between APSIM parameters and population structure is in line with the fact that SNPs with additive effects were sampled at random and some of them had moderate to large Fst (between 0.11 and 0.72 with a median of 0.50).

#### Environment Classification

**Figure 4:**
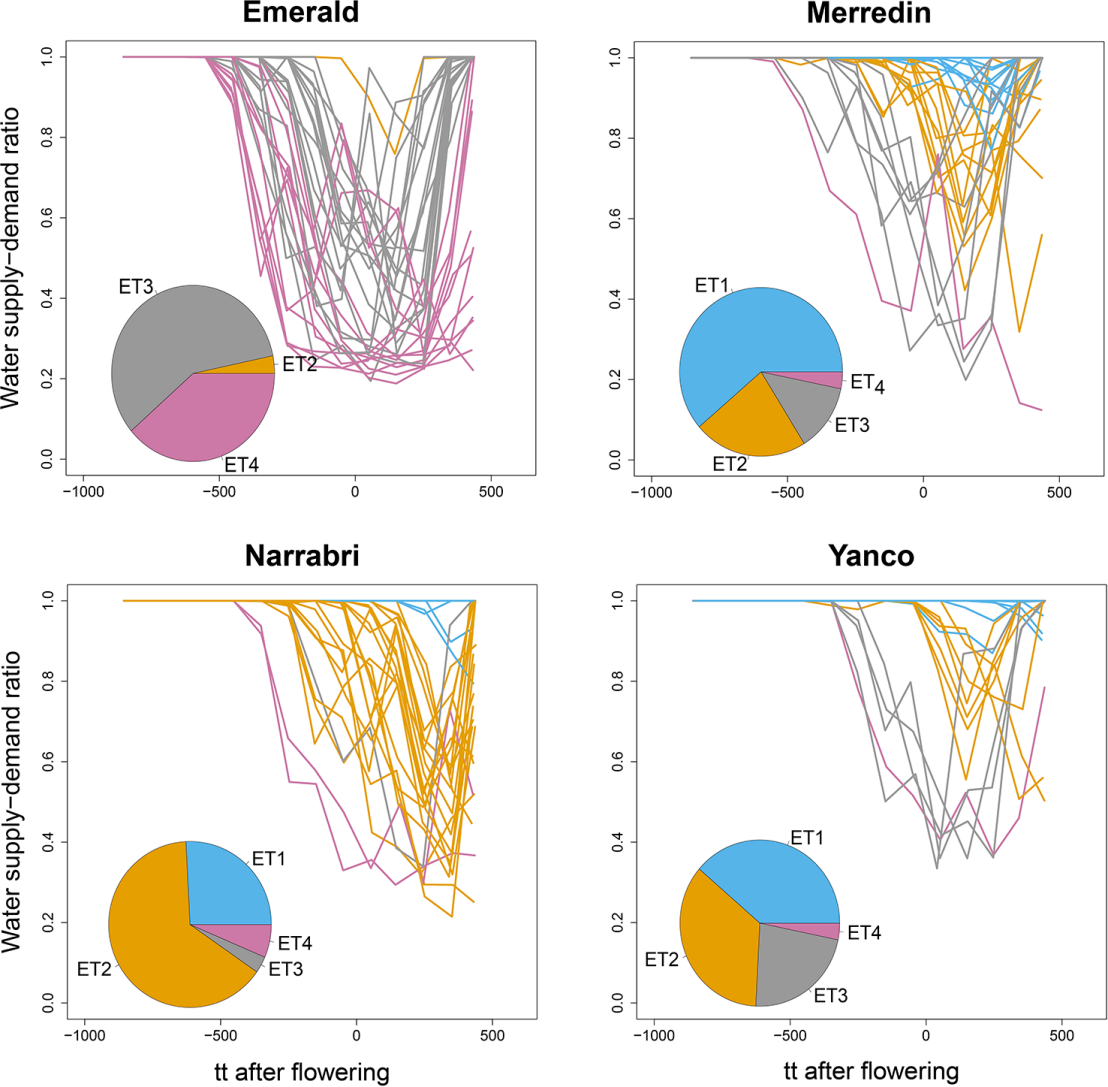
APSIM simulated water supply–demand ratio (stress index) for a genotype with average parameter values growing in Emerald, Narrabri, Merredin, and Yanco during 1983–2013, considering windows of 100^o^Cd. Thermal time was expressed as cumulative degree days from flowering as computed by APSIM. The water supply/demand ratio indicates the degree to which the soil water extractable by the roots (water supply) is able to match the potential transpiration (water demand). The water demand (mm) corresponds to the amount of water the crop would have transpired in the absence of soil water constraint and is estimated on a daily basis from the amount of crop growth on that day (g mm^–2^), and the atmospheric saturation vapor pressure deficit (kPa, Chapman et al., 2000b; Chenu et al., 2011). Line colors correspond to the four environment types obtained by hierarchical clustering of all location × year combinations together. Pie charts represent the frequency of occurrence of environment types at each location for the 31 year period.

We used the water supply-demand ratio over time of a genotype with the average values for the APSIM parameters to classify the 124 environments into four main ETs (**Figure 4**). The combination of the four locations with the series of years is considered to represent well the Australian TPE for wheat. We found in our APSIM simulations that locations largely differed in the frequency with which trials at those locations were assigned to ETs; ET1 (no drought) was most frequent in Yanco and did not occur in Emerald (which has low within-crop rainfall), ET2 (intermediate water stress after flowering, with relief at the end of the growing season) was most common in Narrabri and Yanco, ET3 (strong water stress around flowering, with later relief) and ET4 (very strong water stress starting before flowering with very late or no relief) were most common in Emerald, with a low frequency in the other locations.

#### Representing G×E Patterns With a Sample of the TPE

**Figure 5:**
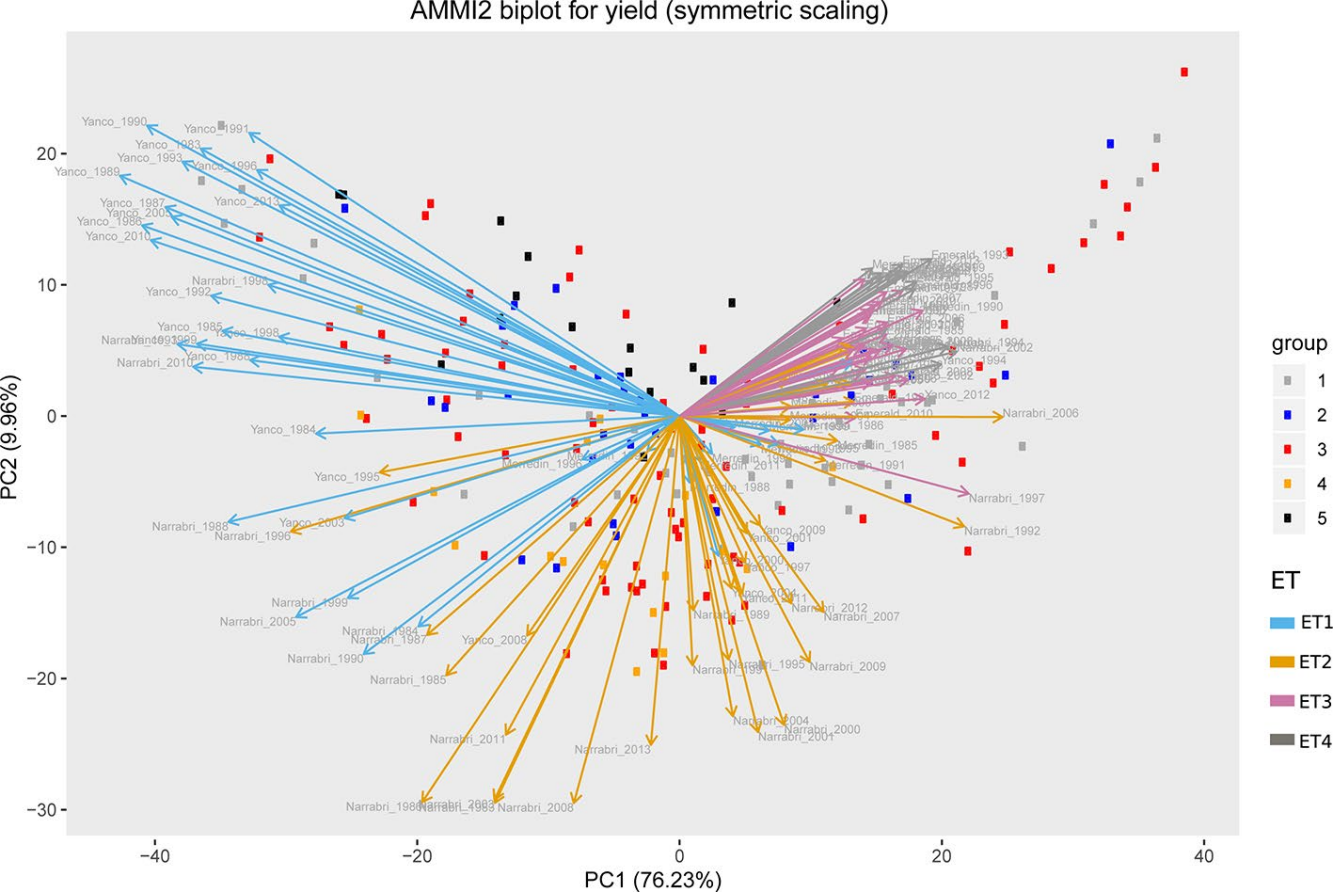
AMMI biplot for simulated grain yield (kg ha^−1^) in Emerald, Merredin, Narrabri, and Yanco during 1983–2013. Circles represent genotype scores (colored by groups) and arrows represent environment scores (colored by environment type).

Simulations generating data for a sample of the TPG and TPE need to be checked for the validity of the generated G×E patterns. Statistical techniques to investigate G×E patterns are fitting AMMI models and estimating variance components for G×E. We also look at the contribution of subsets of genotypes and environments (trials) to simulated G×E. We used the APSIM-simulated yield of the 199 genotypes grown across a large sample of the TPE (4 locations and 31 years, 1983–2013) to identify a subset of trials that represent the most important growing conditions driving G×E. In the simulated TPE sample, G×E for yield was 73% of the total phenotypic variance, whereas G×E for biomass “only” corresponded to 57% of the total phenotypic variance (**Table 4**). For yield, the largest proportion of the G×E was driven by *GLY_ijk_* and *GL_ij_* (45 and 44% respectively), whereas *GY_ik_* explained 11% of the G×E variance. For biomass, the largest proportion (65%) of the G×E was driven by *GL_ij_*, followed by *GLY_ijk_* (27%) and *GY_ik_* explaining 8% of the G×E variance. The G×E patterns observed for yield in the AMMI biplot (**Figure 5**) are closely related to the water deficit dynamics (**Figures 4** and **5**). G×E is driven to a large extent by the contrast between environments without strong water stress (e.g. most of the environments in Yanco) and those that suffer from severe drought starting before flowering (e.g. most of the environments in Emerald and Merredin). Accordingly, the ETs obtained from the water deficit patterns explained a large proportion of G×E variance (57% for yield and 59% for biomass, **Table 5**). The environmental conditions also modified mean yield and biomass across environments. Locations that commonly suffered from drought and that have soils with a lower plant available water capacity, like Emerald and Merredin, showed a lower yield and biomass than Narrabri and Yanco (**Figures S2** and **S3**).

**Table 4:** Variance components and standard error for genotype, genotype by location, genotype by year, and residual G×E, as estimated by mixed model analysis of the APSIM outputs for yield and biomass at maturity of the 199 genotypes in the 124 environments used to characterize the TPE (Emerald, Merredin, Narrabri, and Yanco between 1983 and 2013).

Component	Yield		Biomass	
	Variance	s.e.	Variance	s.e.
Genotype (G*_i_*)	0.056	0.008	0.417	0.051
Genotype.Location (*l_ij_*)	0.067	0.004	0.358	0.021
Genotype.Year (GY*_jk_*)	0.016	0.001	0.045	0.002
Genotype.Location.Year (GLY*_ijk_*)	0.065	0.001	0.151	0.002

**Table 5:** Variance components and standard error for genotype, genotype by environment type, and residual G×E, as estimated by mixed model analysis of the APSIM outputs for yield and biomass at maturity of the 199 genotypes in the 124 environments used to characterize the TPE (Emerald, Merredin, Narrabri, and Yanco between 1983 and 2013).

Component	Yield		Biomass	
	Variance	s.e.	Variance	s.e.
Genotype (G*_i_*)	0.029	0.005	0.306	0.039
Genotype.EnvType (GT*_it_*)	0.086	0.005	0.321	0.019
Genotype.Location.Year (GE(T)_ijk(t)_)	0.066	0.001	0.224	0.002

The complexity of the observed patterns in the simulated G×E and their biologically acceptable interpretations led us to the conclusion that the APSIM generated phenotypes were realistic and useful for testing the performance of statistical models for phenotypic variation under G×E.

### Evaluating Statistical Genotype-To-Phenotype Models That Consider Multiple Traits and Multiple Environments

**Figure 6:**
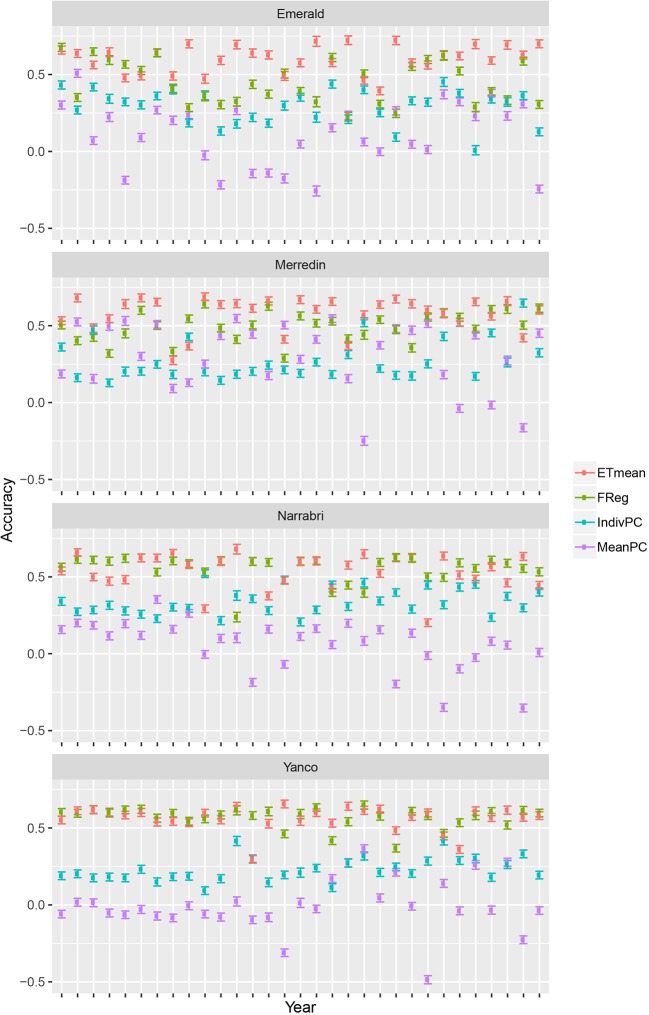
Prediction accuracy and standard error for environments in Emerald, Merredin, Narrabri, and Yanco, between 1983–2013. 20 training sets with four environments randomly drawn from each of the four environment types were used to train the following models: ETmean (yield predicted per environment type, using an unstructured model for environment types), FReg (factorial regression model), IndivPC (independent model terms for each of the cross-products between four genotypic and three environmental scores), MeanPC (mean of the cross-products of genotypic and environment scores).

Crop growth model simulations as done here, can be useful to compare multi-environment genomic prediction models that could later be applied to empirical data. For illustration, we trained a number of models differing in the way that environmental information was incorporated. Although there were some differences in model ranking between environments, in general, the largest prediction accuracy was achieved by the ETmean (with an unstructured variance-covariance for the four ETs, estimated from the simulated yield), emphasizing the interest of grouping environments into more homogeneous classes (**Figure 6**). The second largest accuracy was obtained by the FReg model (APSIM physiological parameters represent the genotypic sensitivity to environmental covariables), showing the importance of variable selection methods in both the genotypic and environmental dimension. After the forward selection procedure, the final factorial regression model considered the basic traits (APSIM parameters with H^2^ of 0.7) of radiation use efficiency (y_rue), sensitivity to vernalization (vern_sens), and sensitivity to photoperiod (photop_sens). The selected environmental covariables were supply–demand ratio during period 3 (sd_P3), sum of radiation in period 4 (radsumP4), maximum photoperiod in period 4 (photomaxP4), mean photoperiod in period 2 (photomeanP2), the mean of radiation during period 2 (radmeanP2), and the sum of radiation in period 4 (radsumP4), as shown in model 17:

(17) $$\begin{array}{c} y_{ij}=\\ \mu +E_{j}+\kappa_{1}y\_rue.sd\_P3_{ij}+\kappa_{2}y\_rue.radsumP4_{ij}+\\ \kappa_{3}vern\_sens.photomaxP4_{ij}+\\ \kappa_{4}photop\_sens.photomeanP2_{ij}+\kappa_{5}photop\_sens.sdP3_{ij}+\\ \kappa_{6}photop\_sens.radmeanP2_{ij}+\\ \kappa_{7}photop\_sens.sdP3_{ij}+\;\kappa_{8}photop\_sens.photomeanP2_{ij}+\\ \;\kappa_{9}y\_rue.sdP3.radsumP4_{ij}+\\ \kappa_{10}photop\_sens.photomeanP2.sdP3_{ij}+\;\varepsilon_{ij} \end{array}$$

The model with estimates for the proportionality constants, *ĸ*_1_ to *ĸ*_10_, to the selected cross-products of genotypic and environmental covariables (IndivPC, using the principal components of the environmental kinship to represent genotypic sensitivities) was clearly better than the MeanPC model (using the mean of the principal components of the environmental kinship to represent genotypic sensitivities) model that avoids any differential weighting and simply includes all available information in the genotypic and environmental dimensions.

### Identifying Traits and Underlying QTLs That Are Useful for Adaptation Across Environments

**Figure 7:**
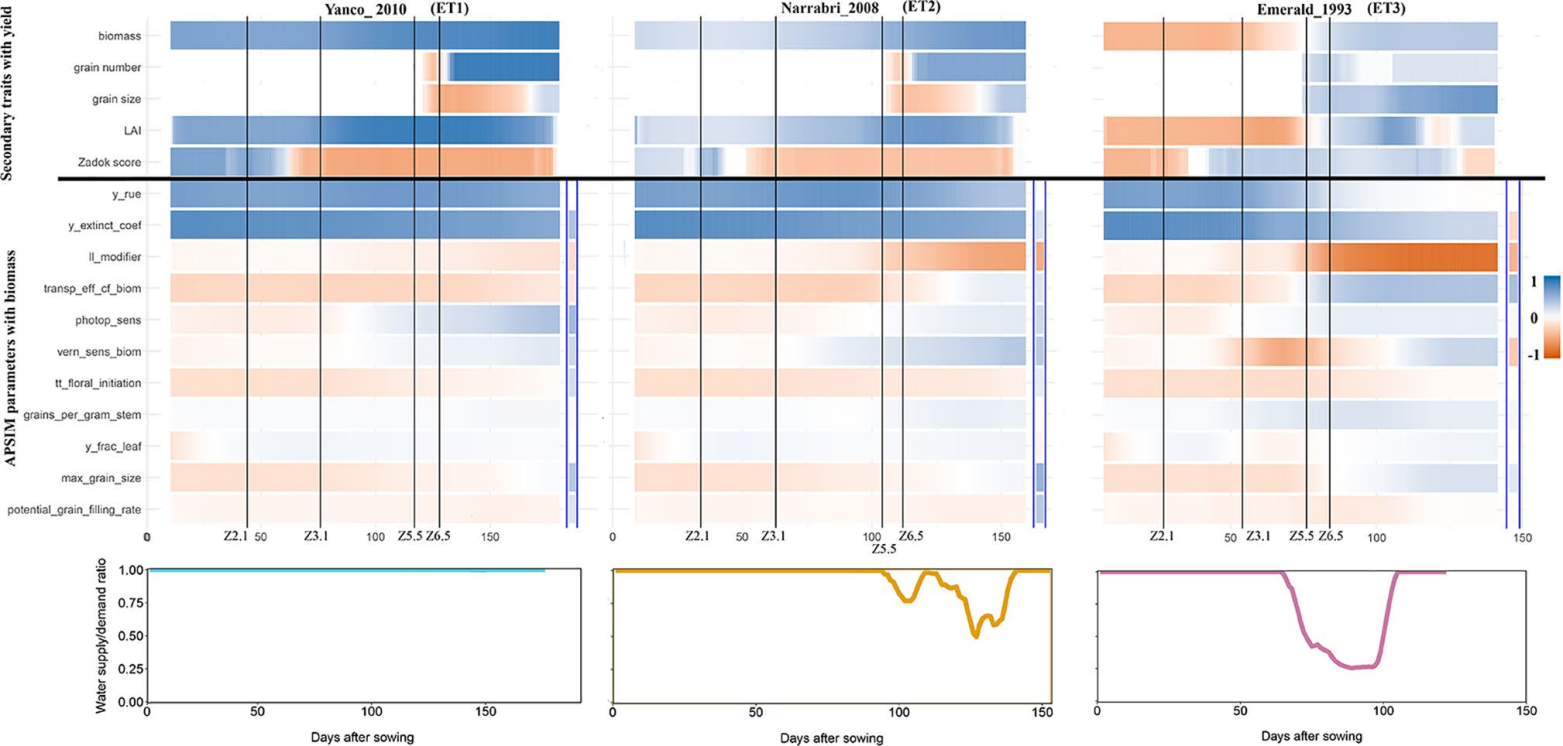
Upper panels: For three location/year combinations, the correlation between daily phenotypes for secondary traits and grain yield at the end of the growing season, and correlation between APSIM parameters (which are a single constant for each genotype) and daily biomass (dark blue indicates a correlation of +1 and dark red indicates a correlation of −1). The correlation between APSIM parameters and grain yield at the end of the growing season is shown between the blue vertical bars. Vertical lines indicate mean phenology for the population, expressed as Zadoks scores for the population mean (Z2.1 is the beginning of tillering, Z3.1 is the beginning of stem elongation, Z5.5 is heading and Z6.5 is anthesis). Lower panels: water supply/demand ratio for the population mean, calculated for sliding windows of 100 ^o^Cd from sowing.

The intensity and temporal dynamics of the correlations between yield and its underlying traits reflect the mechanisms of adaptation and the periods of environmental stress. In our simulations, trait correlations depended on the environmental conditions explored during the growing season. Within a given location, the correlation between biomass and yield (**Figure S6**) and the correlation between Zadok’s development score (**Figure S7**) and yield were heavily influenced by the water deficit patterns (ETs). For a more detailed illustration, we randomly selected three simulated environments that show contrasting water deficit patterns: (a) Yanco_2010 (ET1), (b) Narrabri_2008 (ET2), and (c) Emerald_1993 (ET3). For simplicity, ET4 was not included in the detailed analysis because it induced a similar genotypic response as ET3, when assessing the AMMI biplot. The correlation between biomass and yield differed greatly across the three selected environments and over time (**Figure 7**). Differences in the correlation patterns also coincided with the water deficit dynamics; the correlation between biomass and final yield was intermediate through the season in the late-stress environment Narrabri_2008 and large (>0.8) in the non-stress environment and Yanco_2010. In contrast, the correlation with grain yield in the dry environment Emerald_1993, was negative for biomass at the beginning of the growing season and only became positive after heading.

Simulated data also allows to identify which traits are conferring adaptation to specific environments and help directing selection processes. When descending over the trait hierarchy and focusing on the basic traits, the correlations between APSIM parameters (constant over time for each genotype and across environments) and biomass changed across environments and over time (**Figure 6**). These changes differed between APSIM parameters. For example y_rue (efficiency of conversion of radiation to carbohydrates) and y_extinct_coef (effectiveness of leaf area to intercept radiation) showed little variation over time, indicating that traits related to radiation capture provide a constitutive advantage for biomass accumulation, except for severe water stress. In the dry environment Emerald_1993, the correlation between y_rue, y_extinct_coef, and biomass dropped after heading to become almost zero during the grain filling period. APSIM parameters that are related to water uptake (ll_modifier) and water use efficiency (transp_eff_cf) had a larger impact in the dry environments (**Figure 6**). However, it has to be noted that y_rue and transp_eff_cf were genetically correlated and therefore it is hard to disentangle the effects of each of these parameters on biomass. The correlation between the water-related parameters and biomass was stronger (negative) in dry than in humid environments. This indicates that both ll_modifier (related to effectiveness of root water extraction) and transp_eff_cf (more efficient production of carbohydrates per unit of water transpired) are valuable traits for drought-adapted genotypes (Voltas et al., 1999; Rebetzke et al., 2002; Rebetzke et al., 2012). Phenology-related parameters also changed their correlation with biomass across environments and over time. Photoperiod sensitivity was negatively correlated with biomass before flowering, indicating that more sensitive genotypes (i.e. that flower earlier) also have a smaller biomass accumulation, coinciding with a shorter growing season. During post-flowering, photoperiod sensitivity had a positive correlation with biomass, showing that genotypes can have a larger biomass accumulation during the later stages of the growing season as long as they do not exhaust the water supply in shallow soils with low rainfall (as in Emerald). Vernalization requirements had a stronger negative correlation with biomass in Emerald_1993, than in the other environments. Across environments, the changes of correlations between biomass and final yield, and those between APSIM parameters and biomass coincided with the key phenological stages, especially with heading and flowering time, reflecting the importance of these stages in triggering adaptation processes.

APSIM simulations were also used to explore the impact of basic traits (APSIM parameters) on final yield (**Figure 7**). The correlation between APSIM parameters and final yield was in several cases reversed, compared to that between APSIM parameters and biomass. For example, y_extinct_coef had a positive correlation with biomass in Emerald_1993, but a negative correlation with the final yield, an outcome that can arise when the rapidly growing crop exhausts its water supply around flowering, with consequent decreases in crop growth rate and grain set and yield. The positive correlation was preserved in Narrabri_2008 and Yanco_2010. These sign reversions are a reflection of the propagation of effects through the trait hierarchy.

**Figure 8:**
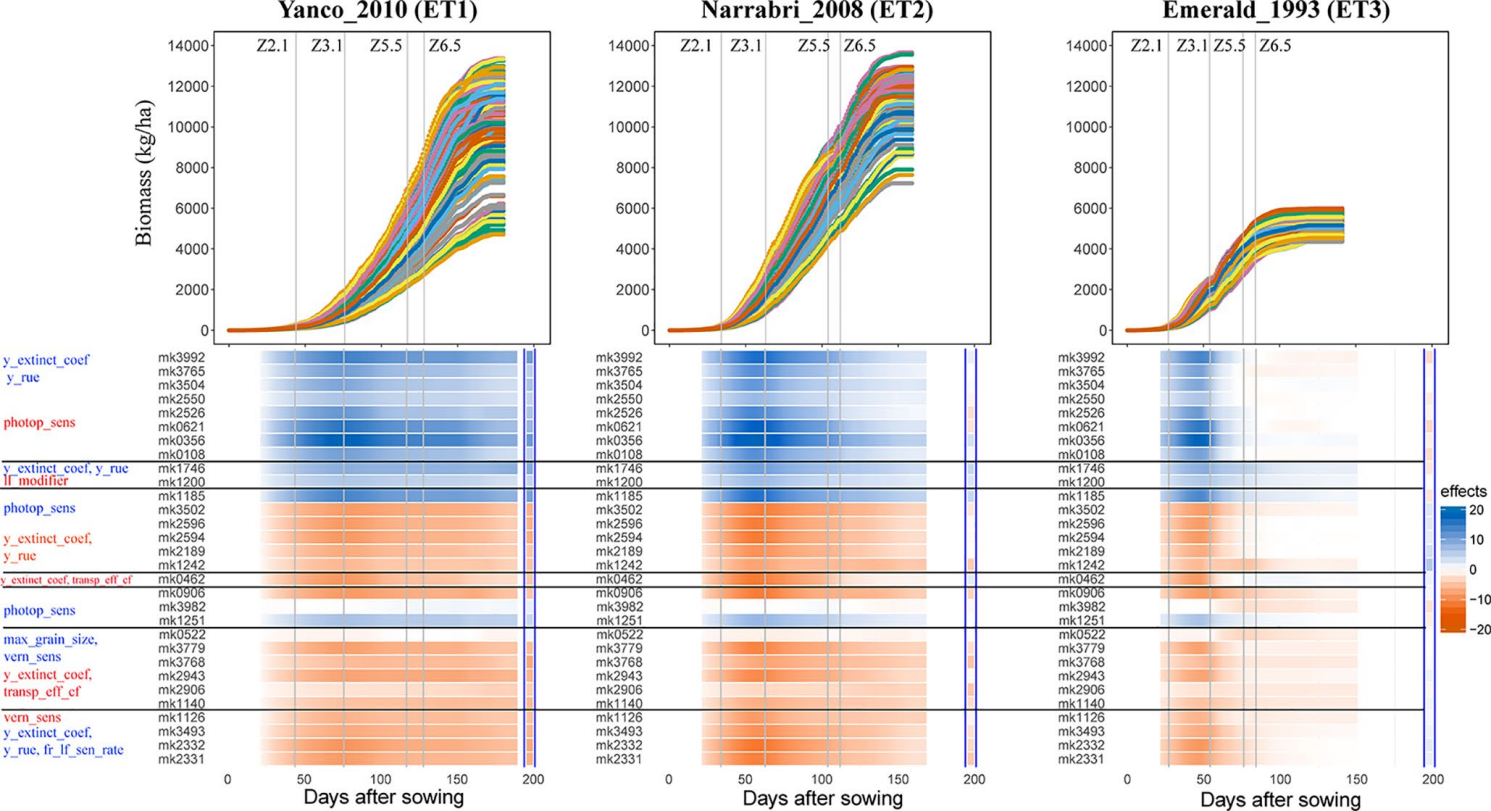
Upper panels: For three location/year combinations, the daily biomass production (kg ha^−1^) for the 199 wheat genotypes. Lower panels: QTL additive effects for biomass estimated from the GWAS for the daily APSIM output. QTL additive effects are expressed as a percentage of the population mean at a given environment and day. SNPs are grouped based on their effects on the APSIM parameters. APSIM parameters written in blue are increased by the most frequent SNP allele, whereas APSIM parameters written in red are decreased by the most frequent SNP allele. Vertical lines indicate mean phenology for the population, expressed as Zadoks scores for the population mean (Z2.1 is the beginning of tillering, Z3.1 is the beginning of stem elongation, Z5.5 is heading and Z6.5 is anthesis). The effect (kg ha^−1^) of the biomass QTLs on final grain yield at the end of the growing season is shown between the blue vertical bars.

Simulations allow analysis of how trait effects are propagated, informing breeders about ET-specific selection of alleles, in a kind of biological sensitivity analysis. We assessed the effects of QTLs for APSIM parameters on daily biomass in the three environments, and grouped the co-localizing QTLs (**Figure 8**). QTLs that had a positive effect on y_extinct_coef and y_rue (mk3992 and mk3765) and a negative effect on photop_sens (mk0621) had a positive effect on biomass in Narrabri_2008 and Yanco_2010. The same QTLs had a positive effect in Emerald_1993 during pre-anthesis and became slightly negative during post-anthesis. This change of the additive effects over time in the dry environment coincides with the temporal change of the phenotypic correlation between biomass and its underlying parameters (**Figure 7**). Other QTLs that also showed strong evidence of G×E were those contributing to increase of photop_sens and ll_modifier. Two of these QTLs (mk3982 and mk0572) were not significant in the non-stress environments, whereas they had a large negative effect in Emerald_1993 during post-anthesis, when water became most limiting. QTL analyses that model G×E in terms of QTL×E can benefit from the insights extracted from the APSIM simulations. Correlations and co-location of QTLs for basic, intermediate, and target traits give insight in which are the physiological mechanisms that are relevant for adaptation to a particular type of environment. It also shows that the effect of a QTL for biomass will also have an effect on yield only if biomass and yield are correlated. For example, mk3992 had a strong positive effect on biomass in both Yanco_2010 and Narrabri_2008 (**Figure 8**). However, this biomass QTL only had a large effect on yield in Yanco_2010 (and not in Narrabri_2008) because the correlation between biomass and yield was larger in Yanco_2010 than in Narrabri_2008. This information can be used to build multi-trait and/or multi-environment models that account for trait- or environment-specific effects. It can also be used to structure the residual genetic variance in QTL detection models, improving the reliability for QTL detection.

### Biomass Auto-Correlation Over Time G×E

To understand genotypic differences in yield as a function of the environmental conditions, we studied the dynamics of underlying intermediate traits like biomass. Examining the autocorrelation in time for biomass may help to identify critical moments during which genotypic changes occur for biomass and consequently yield. The frequency and amplitude of cross-over interactions in intermediate traits as a function of time (lags) influence the degree of success of using early measurements of intermediate traits to improve the predictions for the target trait (**Figure S7**). For our simulated data, biomass autocorrelation was moderate-to-large for all the environments that we analyzed. A reduced auto-correlation became only relevant when considering a lag of 15 days. Autocorrelation was lowest in the early drought-stress environment Emerald_1993 during the first part of the growing season, coinciding with the onset of water stress (**Figures S7** and **7**). In Narrabri_2008, with mild after flowering stress, biomass autocorrelation was reduced after flowering, coinciding with the start of drought stress in this environment. In the non-stressing environment of Yanco_2010, biomass autocorrelations were large and almost constant (around 0.97) during the whole growing season (**Figure S7**), pointing to very low genotype by time interactions. This means that, in Yanco_2010, biomass accumulation curves for all genotypes run almost parallel. Therefore, biomass in the non-stress environment Yanco_2010, measured early during the growing season (e.g. by some phenotyping method), shows promise as a useful trait for final biomass and yield prediction in non-dry environmental conditions. Across the 124 simulated environments, the changes in genotypic ranking for biomass over time led to changes in the variance partitioning. **Figure S5** shows that G×E for biomass is large at the beginning of the growing season (more than 50% of the phenotypic variance), it decreases during the vegetative period (relative to the genotypic main effect), and it increases again around flowering oscillating at around 50% of the phenotypic variance

## Discussion

For a deeper understanding of the mechanisms underlying adaptation, sizeable METs may be required to guarantee that sufficient genotype by environment combinations are covered to train prediction models. In this paper, we generated biologically realistic MET wheat data with G×E and dynamical secondary traits. Our wheat simulations represented a sample from a TPE as evaluated in 124 trials in the Australian wheat belt. We described the structure of G×E using genotypic and environmental covariables, evaluated genotype-to-phenotype models that consider multiple traits over time and multiple environments and characterized the relationships between traits and their underlying QTLs over time and across environments.

### Simulation Settings

#### Genetic Architecture of APSIM Parameters

To check whether the realized correlations between traits were close to the intended correlations, we calculated the Pearson correlation between simulated APSIM parameters. We showed that the way of selecting the SNPs with additive effects influences the correlation (confounding) between traits (APSIM parameters) and population structure. If causal SNPs are sampled at random (equivalent to the neutral genetic architecture proposed by Pérez-Enciso et al., 2017), the correlation between APSIM parameters and structure will vary between one realized MET and the next, depending on the Fst of the SNPs that were used to assign the largest additive effects to. If SNPs with a large Fst are used to assign the largest additive effects to, the APSIM parameters become more correlated to the population structure, but the correlation is more homogeneous across sampling events because we restrict the QTL locations (general discussion in Bustos-Korts, 2017). The larger correlations with population structure reduce the variation in configurations of APSIM parameters that is present in the TPG. The opposite occurs if we attach additive effects to SNPs with low Fst; APSIM parameters will show a lower correlation with population structure and the parameter space will be explored more homogeneously (general discussion in Bustos-Korts, 2017). In this way, simulation settings can be adjusted to regulate the degree of confounding between the causative alleles and population structure, depending on the type of QTL/genomic prediction model that needs to be evaluated. Another alternative is to use the simulations to evaluate how the confounding between causative QTLs and population structure influences QTL detection.

#### Genotypic Variation in APSIM Parameters Modifies G×E

We used the literature as a basis for our selection of parameter ranges. The selection of parameter ranges is one of the most critical steps, having a large impact on the G×E patterns observed in the apsim output. The set of traits and trait ranges that are adaptive across the TPE is defined by the collection of genotypes belonging to the TPG. Flowering time is one of the most important examples of trait ranges that determine the adaptation to the tpe (**Figure S8**); in some environments, only some flowering time values allow successful completion of the growing cycle. Examples and discussions on how the relationship between TPG and TPE relates to trait ranges can be found in Slafer (2003), Slafer et al., (2005), and in Zheng et al. (2012; 2013). In our simulations, we assessed whether the flowering time ranges from the simulations were similar to those observed in the real phenotypic data and adjusted the parameters accordingly. Thus, the sample of the TPG that we constructed matched the phenology that is usually observed in well-adapted genotypes to dry australian environments.

### Representing the TPG Along the TPE

The APSIM crop growth model was used to generate samples from the TPE. Here, we expanded the work previously done by Chenu et al. (2011; 2013) and Casadebaig et al. (2016), through the incorporation of an explicit genetic basis for the APSIM parameters, to evaluate multi-environment quantitative-genetics models. The G×E patterns and partitioning of the phenotypic variance observed for this simulated combination of samples from TPG and TPE were comparable to the ones reported for real field trials (Cullis et al., 2000; Chenu et al., 2011). The large *GLY_ijk_* and the clear impact of water deficit patterns on G×E supports the convenience of focusing on the analysis of ETs (water-deficit patterns) in place of environments defined by years and locations (Chapman et al., 2002a; Chapman, 2008; Chenu et al., 2011; Chenu et al., 2013; Hammer et al., 2014; Watson et al., 2017). Such simulated data are useful to investigate phenotyping and breeding strategies across the Australian TPE. For example, using our simulated data we could evaluate the potential of biomass (or other traits) measured with different high-throughput phenotyping schedules as a correlated trait to improve yield prediction accuracy (companion paper) (Bustos-Korts et al., 2019). The output of our simulations would also allow breaking complex traits into simpler component phenotypes, facilitating the use of marker-assisted and physiological breeding (Ramstein et al., 2018).

The genetic basis that we used for APSIM parameters consisted of exclusively additive effects. However, we did observe nonlinear trait dynamics during the growing season, which result from interactions between physiological mechanisms, called “physiological epistasis” (Cheverud and Routman, 1995; Cooper, 2004). Combining crop growth models with statistical-genetic models showed how biological or physiological epistasis can arise when scaling up from basic traits (APSIM parameters) to traits that show a higher level of integration of biological processes. Biological epistasis takes place when the phenotypic differences among individuals are influenced by other traits via physiological mechanisms (Cheverud and Routman, 1995; Cooper, 2004). If the relationship between the underlying component traits is non-additive, epistatic effects can occur at the phenotypic level of complex traits even if the gene action is purely additive (Holland, 2001; Chapman et al., 2002a; Cooper et al., 2002; Hammer et al., 2006; Technow et al., 2015). For example, all APSIM parameters regulating canopy growth were additive. However, the intermediate trait “green canopy cover” showed a genotype-dependent response that largely changed during the growing season, leading to temporal changes in the genotypic ranking (**Figure 2**).

### Environment Classification

We examined trait correlations over time and the dynamics of biomass QTL effects. We showed that the genetic correlation between traits and the QTL additive effects are time- and environment-dependent. Examining these dependencies gives insight about the physiological mechanisms that confer adaptation to particular environment types and about their genetic regulation. Given that similar environmental conditions induce similar patterns in trait correlations and QTL effects over time, the dynamics of trait correlations and the QTL effects over time can be used to classify environments. Such an approach is increasingly feasible thanks to high throughput phenotyping techniques that allow to monitor multiple traits over time.

### Using Simulated Data to Evaluate Statistical Models for Phenotype Prediction

Most simulation approaches for genotypes across environments involve the use of linear models that might sample from a univariate- or multivariate distribution to directly generate the target trait, without considering its relationships with the underlying traits (Yang et al., 2015). Although they have proven useful to evaluate quantitative genetic models, they might be overly simplistic, as the same kind of model is used to generate the data, and to produce the genomic predictions. Furthermore, linear models cannot incorporate explicit information about the nonlinear dynamics of multiple traits occurring in multiple environments. Therefore, the most important novel aspect of this paper is the simulation of biologically realistic phenotypes for a set of genotypes across a large number of environments using a crop growth model with parameters that have a quantitative genetic basis.

The availability of (simulated) data of a large number of genotypes, environments, and traits over time is a very useful resource to develop and compare genomic prediction models. In this paper, we evaluated four genomic prediction models: (1) using an environmental classification (ETMean), (2) using the individual principal components of the genotypic and environmental kinships (IndivPC), (3) using the mean of the principal components of the genotypic and environmental kinships (MeanPC), and (4) factorial regression models (Freg). All prediction models were used with a training set having environments randomly drawn from each of the four ETs. In general, models that allowed for biological insight (as ETMean and FReg) or allowing for differential influence of the meteorological information on G×E (as IndivPC ) led to greater accuracy, compared to models that do not incorporate biological insight and that assume a similar influence of all environmental covariables on the phenotype (MeanPC). In that sense, our MeanPC model was similar in spirit to the model proposed by Jarquín et al., (2013) and later applied by Malosetti et al., (2016) and Ly et al., (2017). We chose to use an approximation to their model to circumvent the convergence and singularity problems that arose for a large majority of our training sets, showing that although the model proposed by Jarquín et al., (2013) is conceptually appealing, it cannot be easily applied to large-scale data that have large G×E using mixed model software as ASREML-R.

Besides the evaluation of prediction scenarios, long-term phenotypic data, as we produced, also allow forecasting adaptation to future environmental conditions by estimating the probability of a given ET to occur at a particular location (Chenu et al., 2011; Chenu et al., 2013). Forecasting can be further fine-tuned by using information about ENSO events measured early in the growing season (Rimmington and Nicholls, 1993; Zheng et al., 2018). This information can be used to identify the ET for which to make predictions with the model using the ET mean and could also be used in the context of climate change, where more extreme weather scenarios are likely to become more frequent in key producing regions (Watson et al., 2017).

### A New Generation of Crop Growth Models?

In this paper, we used APSIM-wheat, which considers the most important elements to characterize the adaptation landscape in the Australian wheat belt (Chenu et al., 2011; Chenu et al., 2013). Crop growth models can be used with multiple objectives (Chenu et al., 2017). One of them is to assist breeder’s selection decisions by predicting the phenotype of genotypes that are candidate varieties (e.g. Zheng et al., 2013). A second objective is to generate MET data to describe the trait relationships and the adaptation landscape (as we aimed for in this paper). In this second case, the main interest is not on specific genotypes, but on the adequate representation of the variation patterns due to genotypes, environments, traits, and time (genetic correlations across traits and environments over time). Our simulations provided a realistic representation of the Australian wheat belt, considering the most important G×E patterns, and characterizing the dynamics of the most important traits for adaptation to each ET, with assumed underlying QTLs. Therefore, our simulation output could also be useful to design and evaluate high throughput phenotyping schedules and methods (companion paper) Bustos-Korts et al., (2019).

There is a large body of evidence showing that crop growth models are useful to characterize the performance of cropping systems over time (Asseng et al., 1998; Van Ittersum et al., 2003; Holzworth et al., 2014). However, none of them has been properly parameterized in a single set of experiments for a large number of genotypes. In contrast, for most of them, their structure arose by the concatenation of equations estimated in individual experiments, and additional sets of equations (“modules”) were added a posteriori to the original model structure. Although this model building process is adequate for a number of agronomical and breeding applications, these models typically do not account for the correlation structure between APSIM parameters, and it might lead to models that are overly complex for some breeding applications. This increases the amount of phenotypic data and the computational power that is required to fit them to large sets of genotypes and environments.

Although high-throughput phenotyping offers a great opportunity to estimate genotype specific parameters for crop growth models, the structure of crop growth models could be re-thought when aiming at the prediction of phenotypes for a large number of genotypes (Hammer et al., 2019). One way to get a grip on which genotype-to-phenotype dependencies are really necessary for which environments, is to model them via networks (Cooper, 2004). Networks can consider processes belonging to the same level of organization, as genes and gene expression (Mochida et al., 2011; Torres-Sosa et al., 2012; Liseron-Monfils and Ware, 2015), or they can model processes belonging to more than one level of biological organization. Examples of the relationship between QTLs and phenotypes can be seen in Neto et al. (2010), Valente et al., (2010), Wang et al., (2015), and Wang and van Eeuwijk, (2014). Further approaches as directed networks applied to our simulations are explored in Kruijer et al., (2019). Networks are a valuable tool to characterize the dependencies between traits and QTLs. Such networks, applied to large-scale high-throughput phenotyping data over time (or to the output of simulations) would allow us to better understand the conditional dependencies between genotypes, traits, and environments and can be used as a starting point to re-design a new generation of crop growth models that can be more easily used in breeding applications. Deep learning and network methodology can also be extended to consider the time dimension for multiple traits. Although longitudinal networks have shown promise in other fields of science (De Vos et al., 2017), they have not been yet assessed in the context of plant breeding, making them worthwhile for exploration in further research. Our simulated data could be used to evaluate such methodologies.

## Conclusions

We generated realistic phenotypic data by APSIM wheat for multiple dynamical secondary traits and yield. For the simulations, TPE and TPG were inspired by the wheat adaptation landscape along the Australian wheat belt. These simulations were successful in that they reproduced the most important G×E patterns, and described the dynamics for the most important traits for adaptation to each ET, with their underlying QTLs. Our APSIM wheat simulations provide a system that is useful to (1) explore the structure of (simulated) G×E by using models incorporating genotypic and environmental covariables, (2) evaluate statistical genotype-to-phenotype models considering multiple traits and multiple environments, and (3) characterize relationships between traits over time and across environments, as a way to identify traits that could be useful to select for specific adaptation. We provided illustrations of the adaptation landscape across the Australian wheat belt.

## Data Availability Statement

The simulated data supporting the conclusions of this manuscript will be made available by the authors, without undue reservation, to any qualified researcher.

## Author Contributions

DB-K did the simulations, G×E modelling of the simulation output and wrote the first manuscript draft. MM defined the simulation settings and prediction models. KC defined the simulation settings and provided the APSIM simulation files. SC defined the simulation settings. MB provided feedback on the G×E modelling of the simulation output and on the manuscript text. BZ provided input to run APSIM in R. FE defined the simulation settings and prediction models, provided ideas for the general modelling framework and wrote part of the manuscript.

## Funding

This paper received financial support of the European Union’s Seventh Framework Programme (FP7/2007–2013) under the grant agreement no. FP7-613556 (WHEALBI) and from the EU project H2020 817970 (INVITE). DB-K received financial support from Becas Chile (Comisión Nacional de Investigación Científica y Tecnológica, CONICYT, Chile).

## Conflict of Interest

The authors declare that the research was conducted in the absence of any commercial or financial relationships that could be construed as a potential conflict of interest.

## Supplementary Material

Figure S1 Monthly cumulative rainfall (bars) and monthly average temperature (solid line) in the four locations between 1983 and 2013, with years classified into four environment types (ETs), according to their water deficit pattern.

Figure S2 Histograms and correlations for the genotype-dependent APSIM parameters. These parameters were generated from 300 loci with additive effects. We imposed the following correlations on some pairs of parameters: (a) transpiration efficiency coefficient and radiation use efficiency (r=−0.40, blue), (b) number of grains per gram of stem at flowering and maximum grain size (r=−0.50, red), and (c) maximum grain size and potential grain filling rate (r=+0.45, green). Differences between the imposed and realized correlations are product of the sampling process.

Figure S3 Box plots of grain yield (kg ha^−1^) for 199 genotypes grown in a total of 124 environments (Emerald, Merredin, Narrabri, and Yanco during 1983–2013), with environments colored by environment type, related to their water deficit pattern (**Figure 4**).

Figure S4 Biomass for 124 environments (Emerald, Merredin, Narrabri, and Yanco during 1983–2013), coded as per **Figure S3**.

Figure S5 For grain yield (kg ha^−1^), the G×E variance, G variance, and percentage of G×E with respect to the phenotypic variance considering 124 environments (Emerald, Merredin, Narrabri, and Yanco during 1983–2013). Vertical arrows show mean heading date in the population, as observed in Emerald_1993, Narrabri_2008, and Yanco_2010.

Figure S6 Within-environment autocorrelation (lag = 15 days) of biomass in Emerald_1993, Narrabri_2008, and Yanco_2010.

Figure S7 Correlation between yield and biomass (simulated traits) for Emerald, Merredin, Narrabri, and Yanco during 1983–2013. Lines of different colors indicate the correlation for the four ETs. Pie charts represent the frequency of occurrence of environment types at each location.

Figure S8 Correlation between yield and Zadok’s development score (simulated traits) for Emerald, Merredin, Narrabri, and Yanco during 1983–2013. Lines of different colors indicate the correlation for the four ETs. Pie charts represent the frequency of occurrence of environment types at each location.

Table S1 Genetic map.

Table S2 Marker scores used to characterize the 199 wheat genotypes.
