# Supplementary figures and images for: From QTLs to Adaptation Landscapes: Using Genotype-To-Phenotype Models to Characterize G×E Over Time
Source: Front Plant Sci. 2019 Dec 4;10:1540. doi: 10.3389/fpls.2019.01540 (PMC6904366; doi:10.3389/fpls.2019.01540)

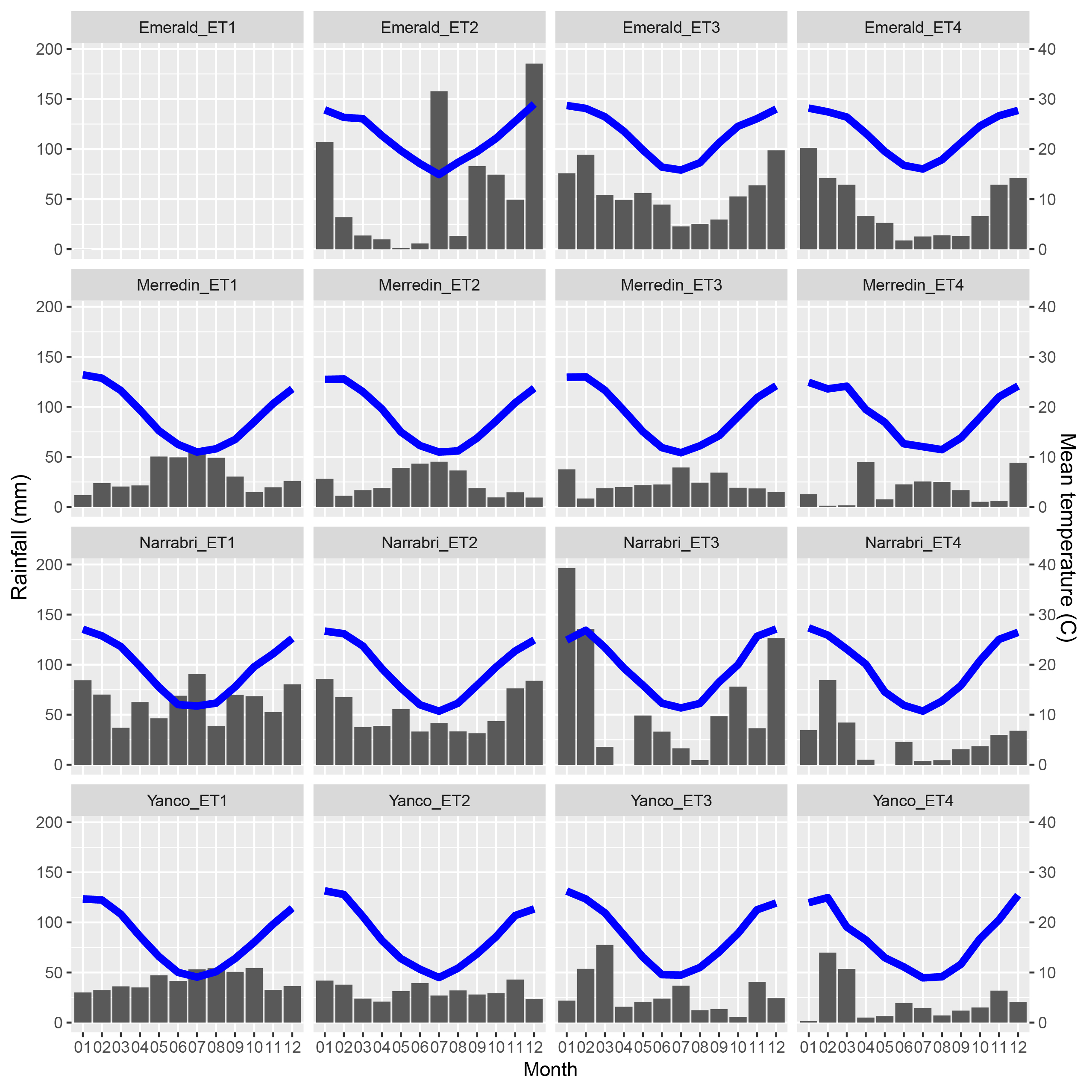

Supplement: Figure S1 — Monthly cumulative rainfall (bars) and monthly average temperature (solid line) in the four locations between 1983 and 2013, with years classified into four environment types (ETs), according to their water deficit pattern. [file Image_1.jpeg]

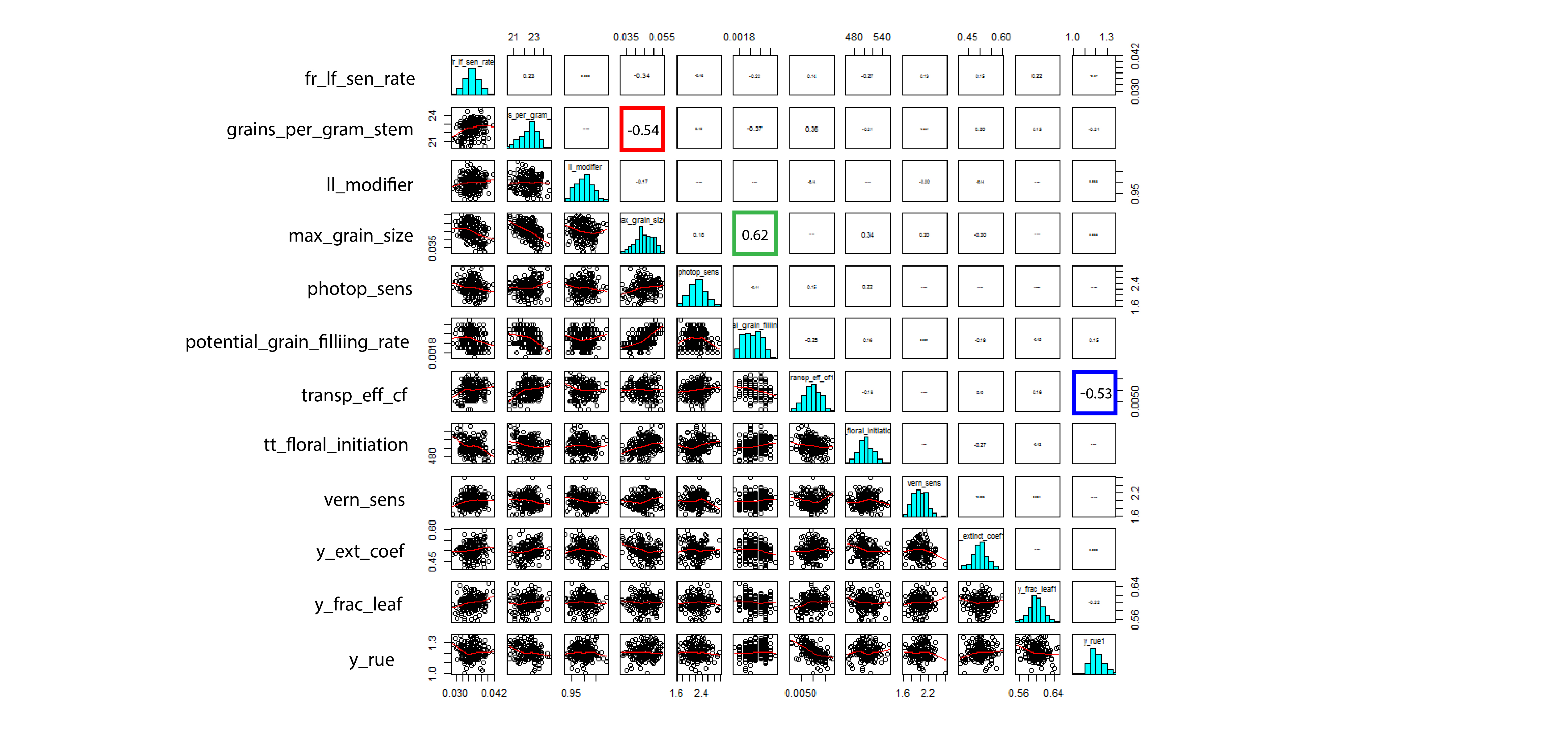

Supplement: Figure S2 — Histograms and correlations for the genotype-dependent APSIM parameters. These parameters were generated from 300 loci with additive effects. We imposed the following correlations on some pairs of parameters: (a) transpiration efficiency coefficient and radiation use efficiency (r=−0.40, blue), (b) number of grains per gram of stem at flowering and maximum grain size (r=−0.50, red), and (c) maximum grain size and potential grain filling rate (r=+0.45, green). Differences between the imposed and realized correlations are product of the sampling process. [file Image_2.jpeg]

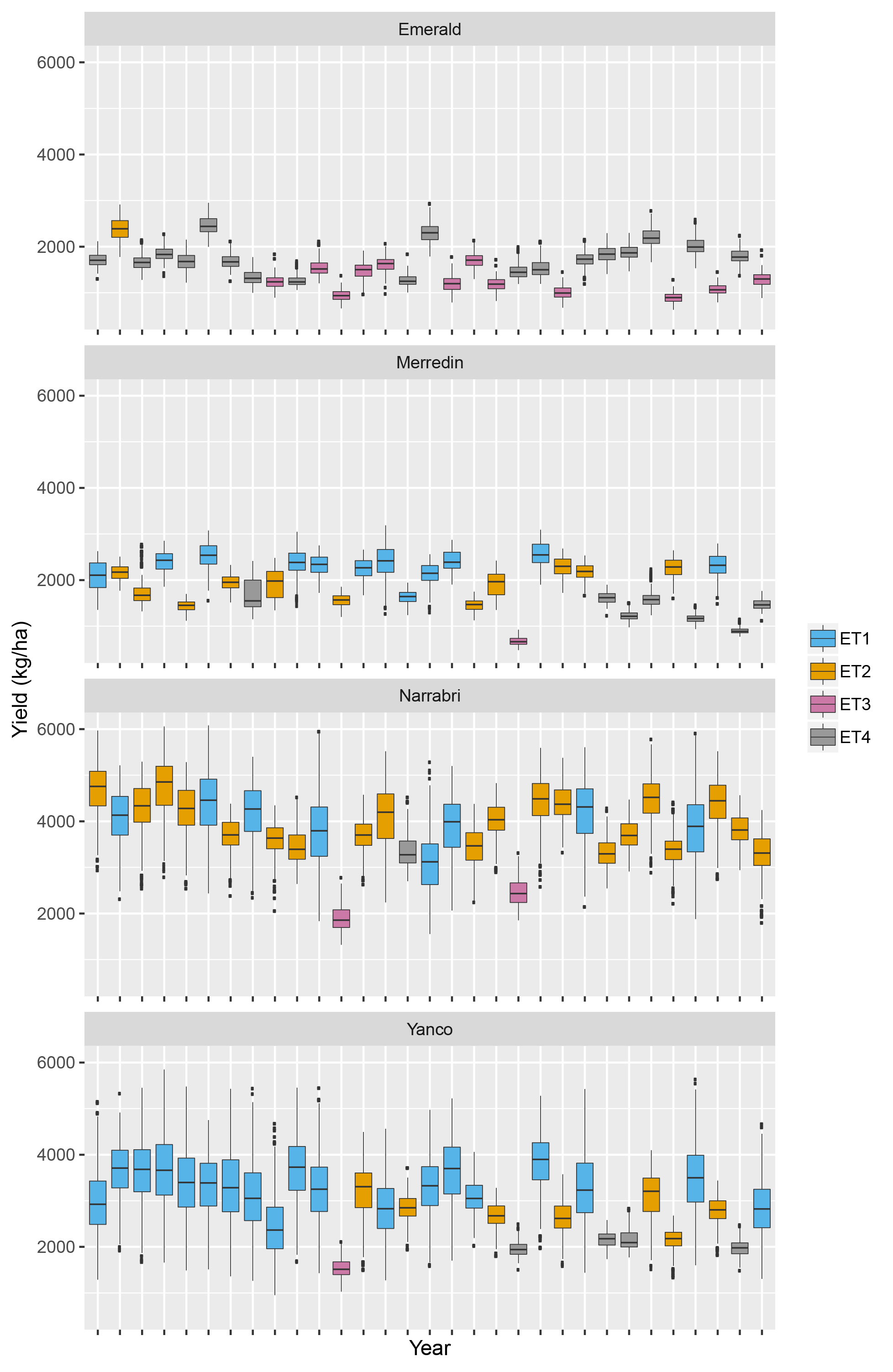

Supplement: Figure S3 — Box plots of grain yield (kg ha−1) for 199 genotypes grown in a total of 124 environments (Emerald, Merredin, Narrabri, and Yanco during 1983–2013), with environments colored by environment type, related to their water deficit pattern (Figure 4). [file Image_3.jpeg]

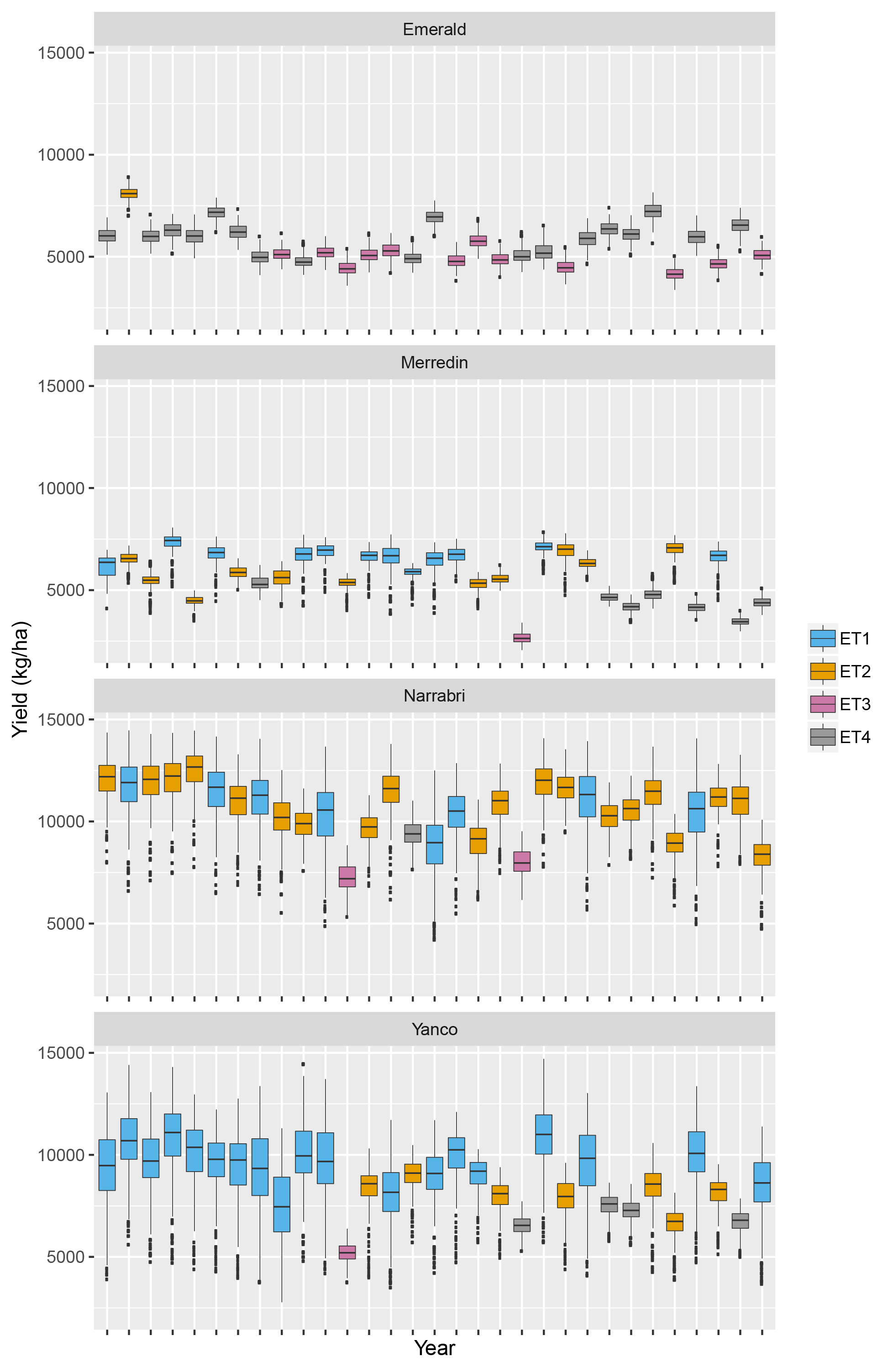

Supplement: Figure S4 — Biomass for 124 environments (Emerald, Merredin, Narrabri, and Yanco during 1983–2013), coded as per Figure S3. [file Image_4.jpeg]

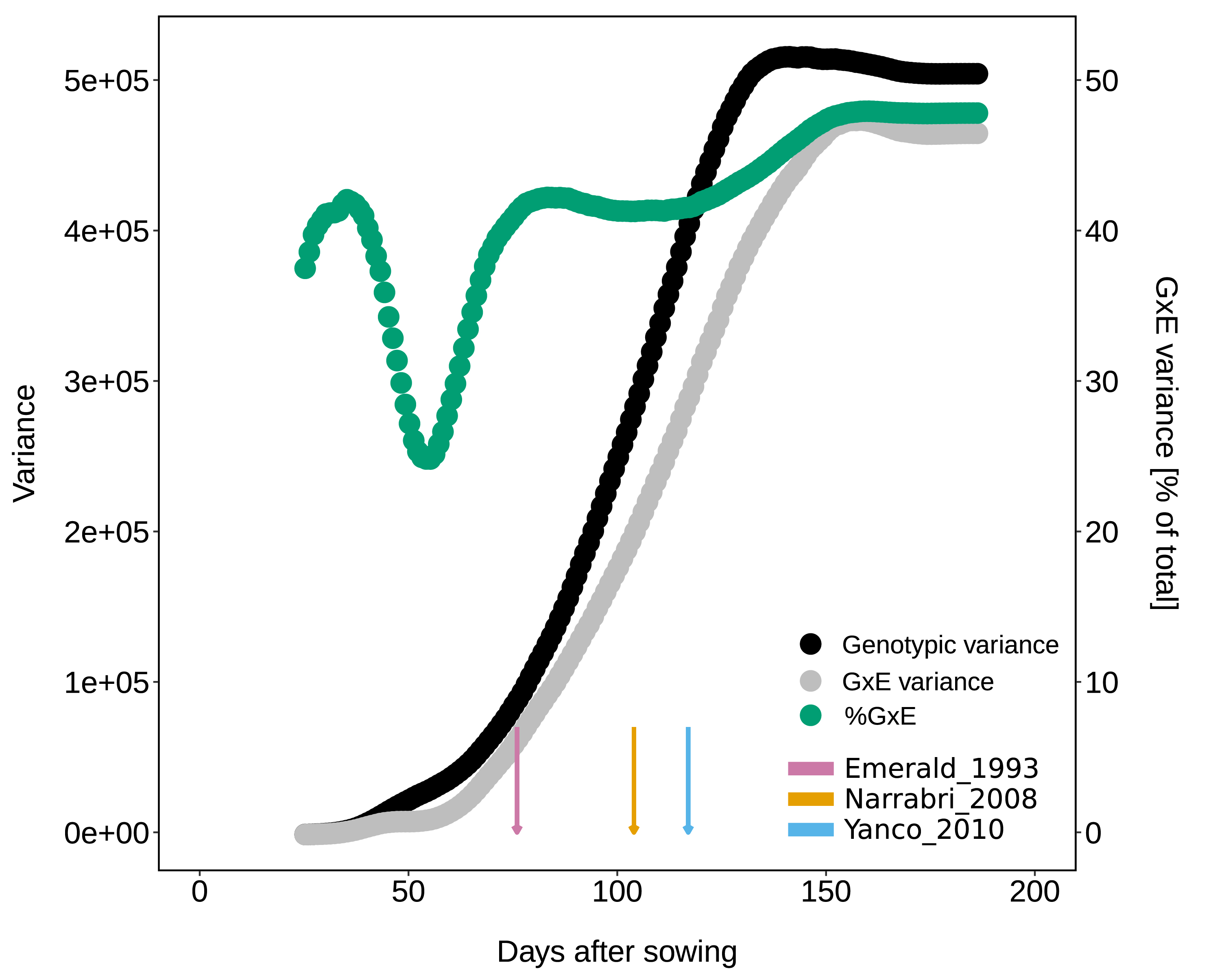

Supplement: Figure S5 — For grain yield (kg ha−1), the G×E variance, G variance, and percentage of G×E with respect to the phenotypic variance considering 124 environments (Emerald, Merredin, Narrabri, and Yanco during 1983–2013). Vertical arrows show mean heading date in the population, as observed in Emerald_1993, Narrabri_2008, and Yanco_2010. [file Image_5.jpeg]

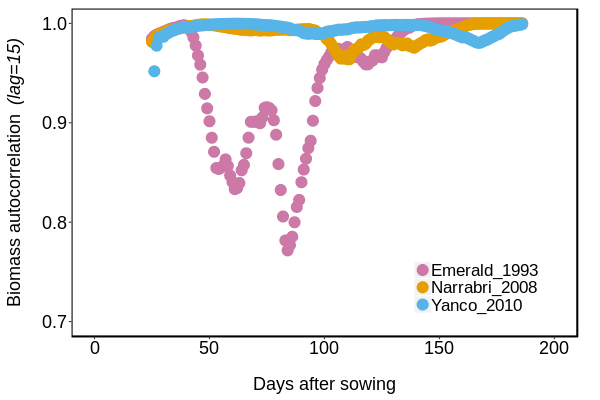

Supplement: Figure S6 — Within-environment autocorrelation (lag = 15 days) of biomass in Emerald_1993, Narrabri_2008, and Yanco_2010. [file Image_6.jpeg]

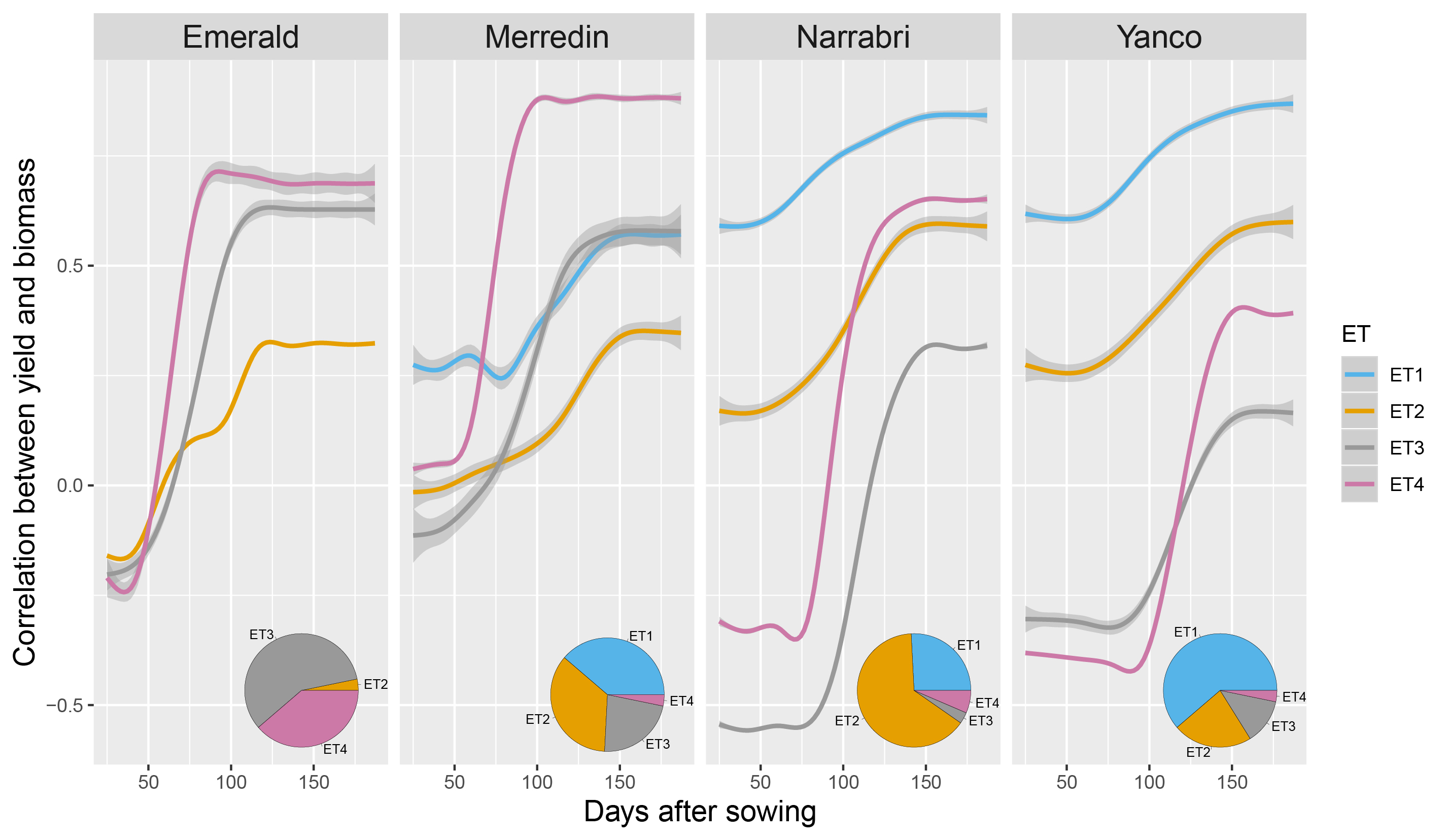

Supplement: Figure S7 — Correlation between yield and biomass (simulated traits) for Emerald, Merredin, Narrabri, and Yanco during 1983–2013. Lines of different colors indicate the correlation for the four ETs. Pie charts represent the frequency of occurrence of environment types at each location. [file Image_7.jpeg]

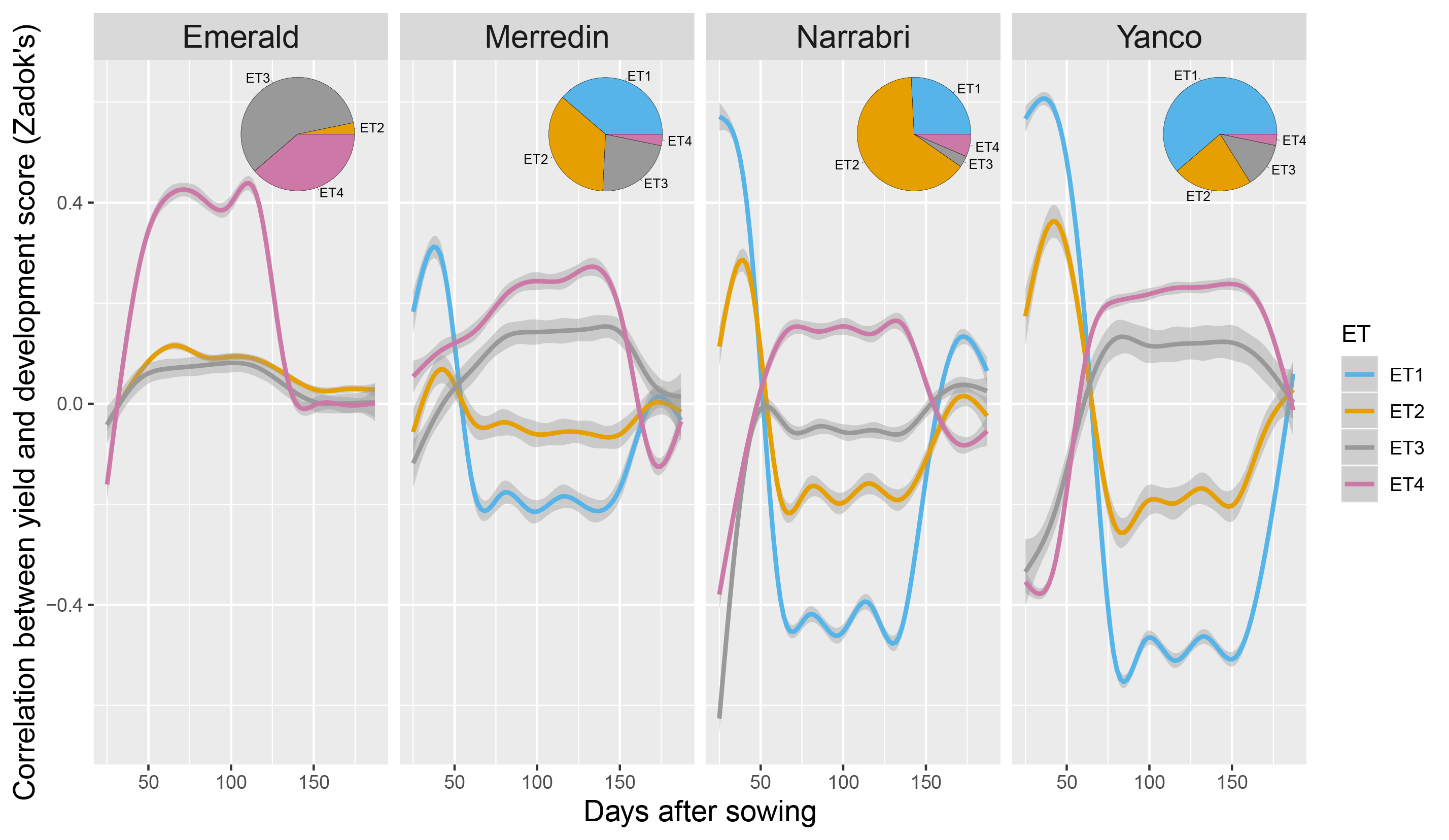

Supplement: Figure S8 — Correlation between yield and Zadok’s development score (simulated traits) for Emerald, Merredin, Narrabri, and Yanco during 1983–2013. Lines of different colors indicate the correlation for the four ETs. Pie charts represent the frequency of occurrence of environment types at each location. [file Image_8.jpeg]
